# Network pharmacology prediction and experimental verification of Rhubarb-Peach Kernel promoting apoptosis in endometriosis

**DOI:** 10.1186/s12906-023-04084-8

**Published:** 2023-08-19

**Authors:** Zi Liao, Ya Lei, Li Peng, Xianyun Fu, Wei Wang, Dan Yang

**Affiliations:** 1 Third-Grade Pharmacological Laboratory On Traditional Chinese Medicine, State Administration of Traditional Chinese Medicine, College of Medicine and Health Sciences, China Three Gorges University, Yichang, China; 2 The First College of Clinical Medicine Science, China Three Gorges University, Yichang Central People’s Hospital, Yichang, China; 3 College of Traditional Chinese Medicine, Three Gorges University & Yichang Hospital of Traditional Chinese Medicine, Yichang, China

**Keywords:** Rhubarb-Peach kernel, Endometriosis, Network pharmacology, Molecular docking, Vitro experiment

## Abstract

**Background:**

“Rhubarb-Peach Kernel” herb pair (RP) one of the most frequently used drug pairs, has been used in traditional medicine in China to treat inflammation and diseases associated with pain. Although it is widely used clinically and has a remarkable curative effect, the mechanism of RP treatment for endometriosis (EMs) remains unclear due to its complicated components. The aim of this study was to investigate the anti-endometriosis effect of RP, with emphasis on apoptosis via network pharmacology prediction, molecular docking and experimental verification.

**Methods:**

The related ingredients and targets of RP in treating EMs were screened out using Traditional Chinese Medicine Systems Pharmacology (TCMSP), Tool for Molecular mechanism of Traditional Chinese Medicine (BATMAN-TCM), and GeneCards database. The data of the protein–protein interaction (PPI) network was obtained by the Search Tool for the Retrieval of Interaction Gene/Proteins (STRING) Database. The Metascape database was adopt for Kyoto encyclopedia of genes and genomes (KEGG) enrichment analysis. After that, the molecular docking of the main active ingredients and apoptosis targets was performed. Finally, the pro-apoptotic effect of RP was verified in hEM15a cells.

**Results:**

A total of 32 RP compounds were collected. Forty-two matching targets were picked out as the correlative targets of RP in treating EMs. Among these, 18 hub targets including P53, CASP3 were recognized by the PPI network. KEGG enrichment analysis discovered that the regulation of apoptosis was one of the potential mechanisms of RP against EMs. Anthraquinone compounds, flavonoids, and triterpenes in RP were identified as crucial active ingredients, involved in the pro-apoptotic effect, which were confirmed subsequently by molecular docking. Additionally, it was verified that RP treatment promoted apoptosis and inhibited the proliferation of EMs cells (assessed by MTT and Flow cytometry). Moreover, the induction of apoptosis in treated EMs cells may be due to the regulation of apoptosis-related protein expression, including P53, BAX, and CASP3.

**Conclusions:**

The results of our study demonstrated that RP may exert its therapeutic effects on EMs through the potential mechanism of promoting apoptosis. Anthraquinones, flavonoids and triterpenoids are the possible pro-apoptotic components in RP.

**Supplementary Information:**

The online version contains supplementary material available at 10.1186/s12906-023-04084-8.

## Introduction

Approximately 5–20% of women of their reproductive age are diagnosed with endometriosis (EMs) [1], which is responsible for agonizing pelvic pain and low fertility with a great impact on the patient's quality of life [2–5]. The pathological manifestation of EMs is characterized by extra-uterine growth of endometrial tissues. Apoptosis and proliferation imbalance [6–8], endocrine tolerance [9, 10], oxidative stress [11, 12], inflammation and immunity [13] and their interaction contribute to the high invasive ability of EMs cells. Although several medication lines including surgery, non-steroidal anti-inflammatory drugs, and hormones have been studied aiming at its definitive treatment, these therapies are difficult to avoid a range of side effects and high recurrence rates [14, 15]. As a result, half of the patients are dissatisfied with the existing medical support [16]. Therefore, it is necessary to find an alternative approach.

The “Rhubarb-Peach kernel” herb pair (RP) is a core medical pair of classical disperse stasis formula and consists of two TCM drugs: *Rheum palmatum L.*, Rhizome and root (R, dahuang), and *Prunus persica (L.) Batsch*, seed (P, taoren). In traditional Chinese medicine, RP has been utilized for the treatment of pain-related disorders. In the case of EMs, it is frequently administered to non-pregnant patients to alleviate symptoms of dysmenorrhea and effectively minimize the incidence of endometrial lesions. The voucher specimen (CNPC2009) of the two plants has been gathered and favorited in the Chinese Field Herbarium for our identification. RP was prepared with the following steps: The pretreated Rhubarb and Peach Kernel were extracted by twice heating water, and then the extracts were combined and filtered. After that, it is vacuum concentrated into a liquid extract with a specific gravity of 1.20–1.35. Finally, spray-dried the liquid extract into granules. Studies have shown that the pharmacological action of RP may relate to proliferation inhibition, apoptosis promotion, oxidative stress improvement, and estrogen effect regulation [17–19]. However, the mechanism of RP treatment for EMs remains unclear due to its complicated components. Nowadays, with the development of network pharmacology and molecular docking technology, many systematic platforms are provided for exploring the mechanism of TCM, which can comprehensively evaluate the pharmacological effects of multiple components, multiple targets, and multiple pathways of herbs [20].

**Fig. 1:**
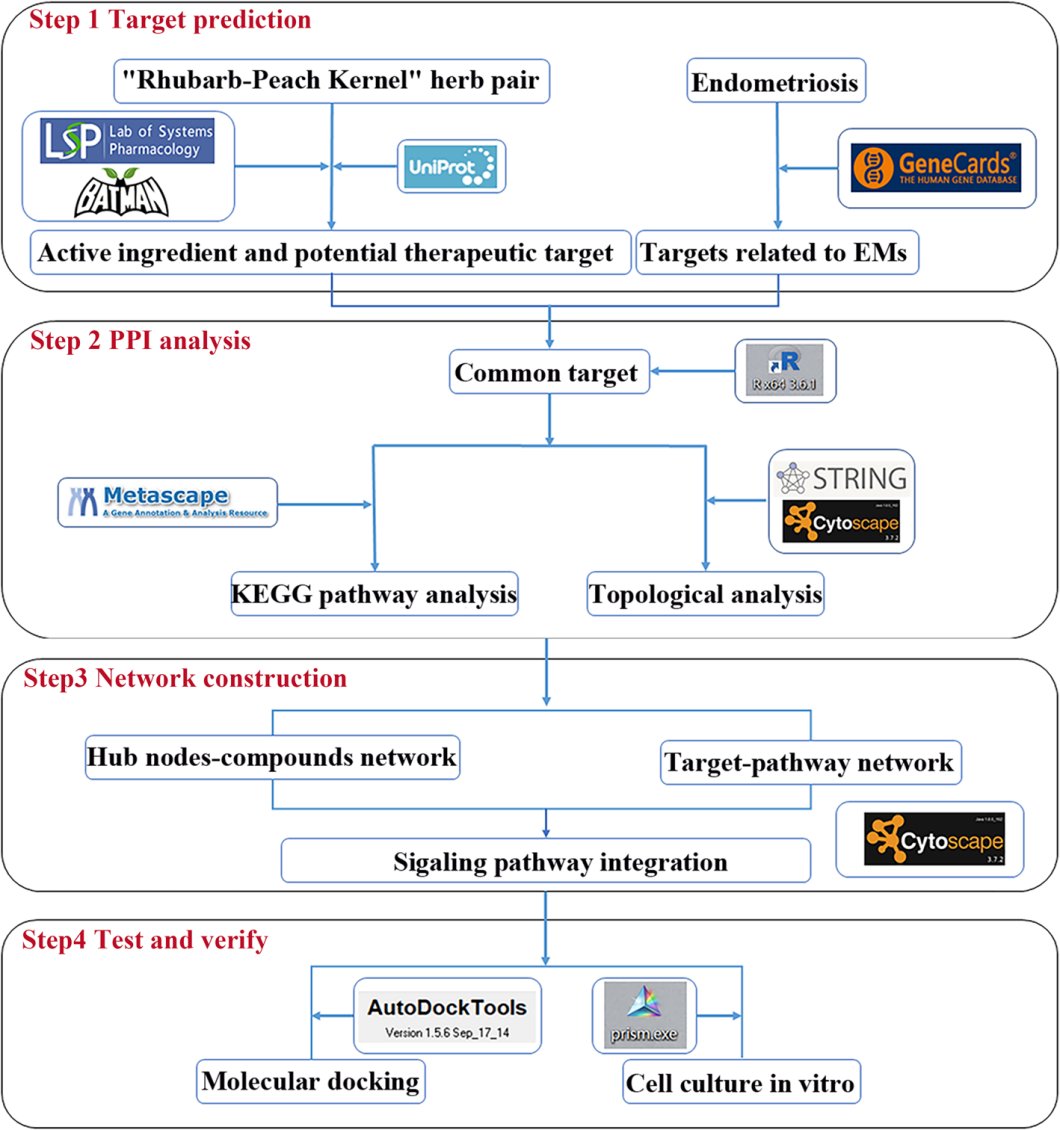
The framework based on an integration strategy of network pharmacology

Therefore, in this study, we aim to investigate the key active components of RP and their potential mechanisms for EMs treatment by network pharmacology prediction, molecular docking and experimental verification. The framework of the research is shown in Fig. 1.

## Materials and methods

### Screening of active ingredients and targets of RP

The Traditional Chinese Medicine Systems Pharmacology Database Analysis Platform (TCMSP, https://tcmspw.com/tcmsp.php) [21] and the Bioinformatics Analysis Tool for Molecular Mechanism of Traditional Chinese Medicine (BATMAN-TCM, https://bionet.ncpsb.org/batman-tcm/) [22] were adopted to search for the active ingredients. Oral bioavailability (OB) ≥ 30% and Drug-likeness (DL) ≥ 0.18 were used as the threshold value to screen out the potential active ingredients [23]. Potential protein targets of the active ingredients were collected through the TCMSP database, then the related gene targets were obtained via UniProtKB of the protein database (https://www.uniprot.org/uniprot/).

### Searching for potential anti-EMs targets of RP

In the GeneCards database (https://www.genecards.org/) [24] and OMIM database (https://www.omim.org/) [25], "endometriosis" was used as the keyword to collect the relevant disease targets of EMs. Then the overlapping targets of RP and EMs, which were considered potential targets of RP in the treatment of EMs, were identified by R3.6.1 software.

### Constructing PPI network

The protein data of overlapping targets were imported into the Search Tool for the Retrieval of Interaction Gene/Proteins database (STRING, https://string-db.org/) [26]. The confidence was set to greater than 0.7 and a protein–protein interaction (PPI) network of the targets was obtained.

### Topological analysis

The PPI network diagram is exported in TSV file format and imported into the visualization software Cytoscape 3.7.2 [27]. “Degree Centrality (DC)” and “Betweenness Centrality (BC)” were selected to evaluate the topological characteristics of the network.

### KEGG enrichment analysis

Metascape database was chosen to perform the Kyoto encyclopedia of genes and genomes (KEGG) enrichment analysis (https://metascape.org/) [28]. With the *P*-value < 0.05, we screened out the main pathways of clustering and sorting.

### Network construction and analysis

The screened active components and their corresponding targets were imported into Cytoscape 3.7.2 to construct a “component-target” network. The KEGG mapper tool in the KEGG database (https://www.kegg.jp/) was used to retrieve the significant pathways corresponding to the target to build the “target-pathway” network. Then, the two networks were integrated into the final “component-target-pathway” network.

### Test and verify

#### Molecular docking

The RCSB Protein Data Bank (PDB, https://www.rcsb.org/) was used to search the 3D structure files of the active components. The 3D structure files of target proteins were obtained through the PubChem (https://pubchem.ncbi.nlm.nih.gov/) and ZINC (https://zinc.docking.org/) databases. Autodock (https://autodock.scripps.edu/) molecular docking software was adopted to dock active compounds with target proteins. The binding sites were analyzed by PyMOL software. The binding ability between them was evaluated by binding energy, and a value ≤ -5 kcal/mol was considered as tight binding.

#### Cells culture

The hEM15a cells provided by China Center for Type Culture Collection were cultured in DMEM/F12 medium supplemented with 10% FBS and 1% penicillin/streptomycin. The cell incubator was maintained at 37 °C with a humid atmosphere containing 5% CO_2_. After that, the vimentin staining of cells was performed by immunofluorescence.

#### MTT colorimetric assay

The hEM15a cells were seeded into a 96-well plate, and incubated at 37 °C with a humid atmosphere containing 5% CO_2_ for 24 h and 48 h respectively after treating with different concentrations of RP (0, 1, 3, 5, 7, 9 mg/ml, consisting of rhubarb and peach kernels at the ratio of 1: 1, Beijing Kangrentang Pharmaceutical Co. Ltd., Beijing, China, NO 19004961, 19006411). The high performance liquid chromatography system was used for the chemical component identifications of RP extract (Supplementary Material S1). Then, the cells reacted with 10 μl MTT for 4 h at 37 °C. After that, 150 μl of DMSO was added, and the absorbance was measured at 568 nm by a microplate reader.

#### Apoptosis analysis

The hEM15a cells were divided into negative control group, control group, and RP group (3 mg/ml). Then, the apoptosis of hEM15a cells was evaluated. After the cells were suspended in 500 μL binding buffer, annexin V-PI solution was added and cultured for 5—15 min without light (negative control was normal cells without annexin V-PI solution). After that, the percentage of apoptotic cells was detected on a flow cytometer.

#### Western blotting

The total protein of hEM15a cells was extracted with 120 μL RIPA buffer containing PMSF and phosphatase inhibitors. Then, it was resolved by polyacrylamide gel electrophoresis and transferred to polyvinylidene fluoride membranes, which were blocked overnight in TBST containing 5% skim milk powder. Primary antibodies against the following proteins were used: P53 (1: 1000, Proteintech Group, Inc, 10442-1-AP,), BAX (1: 1000, Abcam, Ab32503), CASP3 (1: 2000, Abcam, Ab184787), and GAPDH (1: 1000, Hangzhou Xianzhi Biology Co., Ltd, AB-PR 001). The blots cut prior to hybridisation with antibodies. Subsequently, the matched secondary antibody was added to the membrane, and BandScan was used to analyze the gray value of the films.

### Statistical analysis

All results were expressed as mean ± standard. IBM SPSS 20.0 software was used for one-way analysis of variance test for statistical analysis. Differences between groups were considered to be statistically significant if the *P*-value were less than 0.05.

## Results

### Active compounds of RP

According to the retrieval of TCMSP and BATMAN-TCM database, there are 157 RP-related ingredients in total, including 92 ingredients in Rhubarb and 66 ingredients in Peach kernel. Combined with screening criteria (OB ≥ 30%, DL ≥ 0.18) and literature retrieval, a total of 32 active ingredients in RP were obtained, as shown in Table 1.

**Table 1:** Main active ingredients in RP (Arrange by OB value from high to low)

Herb Name	Mol ID	Molecule Name	Code Name	Structure	OB (%)	DL
Rhubarb	MOL000471	aloe-emodin	R-1		83.38	0.24
	MOL002235	eupatin	R-2		50.8	0.41
	MOL000096	(-)-catechin	R-3		49.68	0.24
	MOL002268	rhein	R-4		47.07	0.28
	MOL002281	toralactone	R-5		46.46	0.24
	MOL002288	emodin-1-o-beta-d-glucopyranoside	R-6		44.81	0.8
	MOL002280	torachrysone-8-o-beta-d-(6'-oxayl)-glucoside	R-7		43.02	0.74
	MOL002259	physciondiglucoside	R-8		41.65	0.63
	MOL000358	beta-sitosterol	R-9		36.91	0.75
	MOL002297	daucosterol_qt	R-10		35.89	0.7
	MOL000472	emodin	R-11		24.4	0.24
	MOL000476	physcion	R-12		22.29	0.27
	MOL001729	crysophanol	R-13		18.64	0.21
Peach Kernel	MOL001351	gibberellin a44	P-1		101.61	0.54
	MOL001353	gibberellin a60	P-2		93.17	0.53
	MOL001349	4a-formyl-7alpha-hydroxy-1-methyl-8-methylidene-4aalpha,4bbeta-gibbane-1alpha,10beta-dicarboxylic acid	P-3		88.6	0.46
	MOL001344	gibberellin a122-isolactone	P-4		88.11	0.54
	MOL001329	2,3-didehydro gibberellin a77	P-5		88.08	0.53
	MOL001360	gibberellin a77	P-6		87.89	0.53
	MOL001340	gibberellin a120	P-7		84.85	0.45
	MOL001339	gibberellin a119	P-8		76.36	0.49
	MOL001358	gibberellin 7	P-9		73.8	0.5
	MOL001342	gibberellin a121-isolactone	P-10		72.7	0.54
	MOL001361	gibberellin a87	P-11		68.85	0.57
	MOL001355	gibberellin a63	P-12		65.54	0.54
	MOL001352	gibberellin a54	P-13		64.21	0.53
	MOL001328	2,3-didehydro gibberellin a70	P-14		63.29	0.5
	MOL001323	sitosterol alpha1	P-15		43.28	0.78
	MOL001368	3-o-p-coumaroylquinic acid	P-16		37.63	0.29
	MOL000493	campesterol	P-17		37.58	0.71
	MOL000296	hederagenin	P-18		36.91	0.75
	MOL000358	beta-sitosterol	P-19		36.91	0.75
	MOL001320	amygdalin	P-20		4.42	0.61

### Prediction of potential targets for RP in treating EMs

**Fig. 2:**
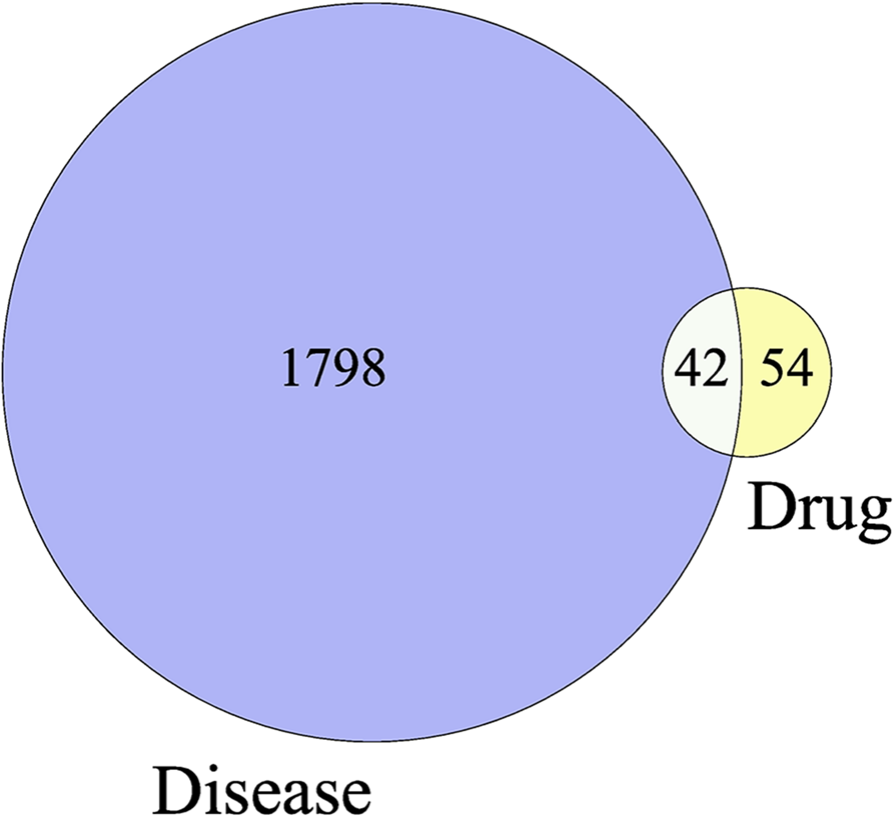
The Venn diagram of the targets both in EMs targets and RP targets

After searching, 32 active ingredients correspond to 96 protein targets. A total of 1840 disease targets of EMs were obtained through the GeneCards database and OMIM database. Then, 42 overlapping targets were obtained, which were considered potential targets for RP in treating EMs, as shown in Fig. 2.

### The interaction of protein targets

**Fig. 3:**
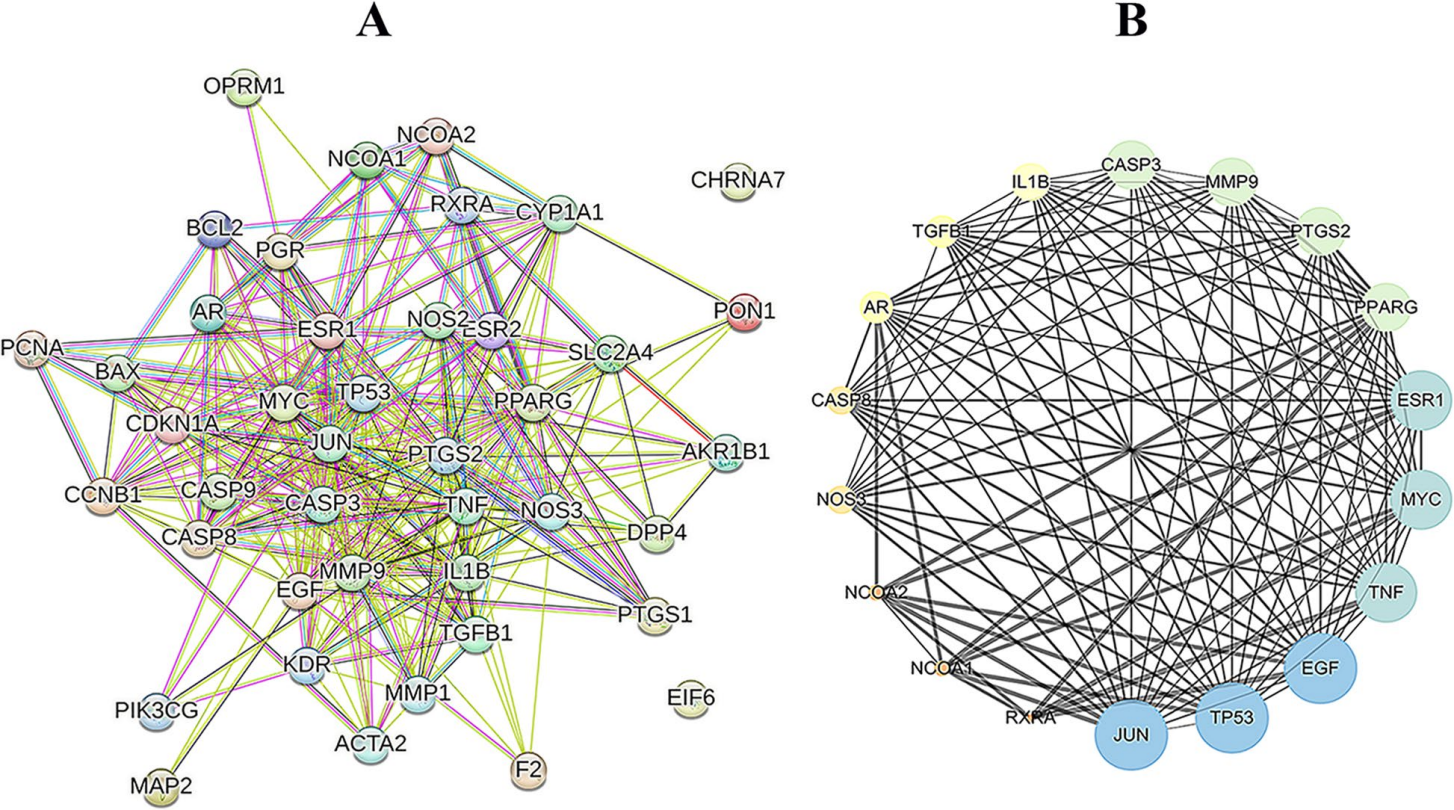
PPI network. **A** The interactive PPI network was obtained from the STRING database. It contains 42 nodes and 319 edges. **B** Hub-gene interactions in the PPI network: 18 significant hubs according to the degree value. The size and color of the nodes represent the degree value (yellow-blue indicates the degree value is low to high), and the size of the edges represents the betweenness value (low value represents small size)

Through the STRING database, a PPI network of those 42 overlapping targets was obtained. With confidence ≥ 0.7, DC ≥ 8 and BC ≥ 0.012948, the PPI network with 18 nodes and 115 edges was obtained. The average node degree was 12.778 and the enrichment *P*-value < 1.0e-16. The nodes included JUN, TP53, EGF, TNF, MYC, ESR1, PPARG, PTGS2, MMP9, CASP3, IL1B, TGFB1, AR, CASP8, NOS3, NCOA2, NCOA1, and RXRA, as shown in Fig. 3.

### Pathway prediction

**Fig. 4:**
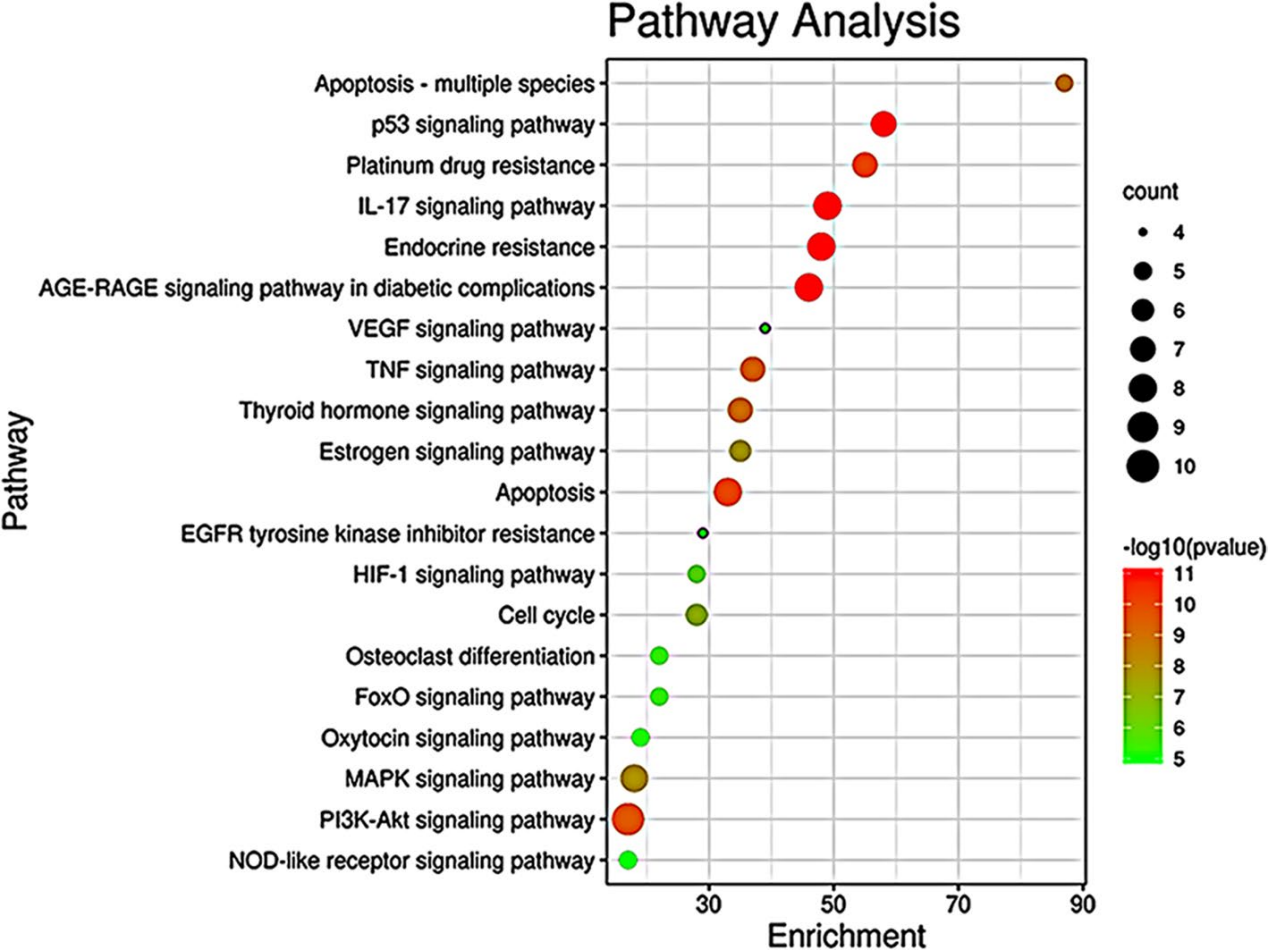
Top 20 KEGG signal pathways. The X-axis represents the enrichment value and the Y-axis shows significantly enriched KEGG pathways of the target genes. Higher enrichment values represent a higher level of enrichment. The size of the dot indicates the number of target genes in the pathway, and the color of the dot reflects the different *P* value ranges

On the Metascape platform, KEGG enrichment analysis was performed [29], and 39 pathways were screened out according to *P*-value ≤ 0.05, such as IL-17 signaling pathway (hsa04657), endocrine resistance (hsa01522), P53 signaling pathway (hsa04115), apoptosis (hsa04210), PI3K-Akt signaling pathway (hsa04151), TNF signaling pathway (hsa04668), MAPK signaling pathway (hsa04010), estrogen signaling pathway (hsa04915), Cell cycle (hsa04110), as shown in Fig. 4.

### Network construction and analysis

#### “Component-Target” network construction

**Fig. 5:**
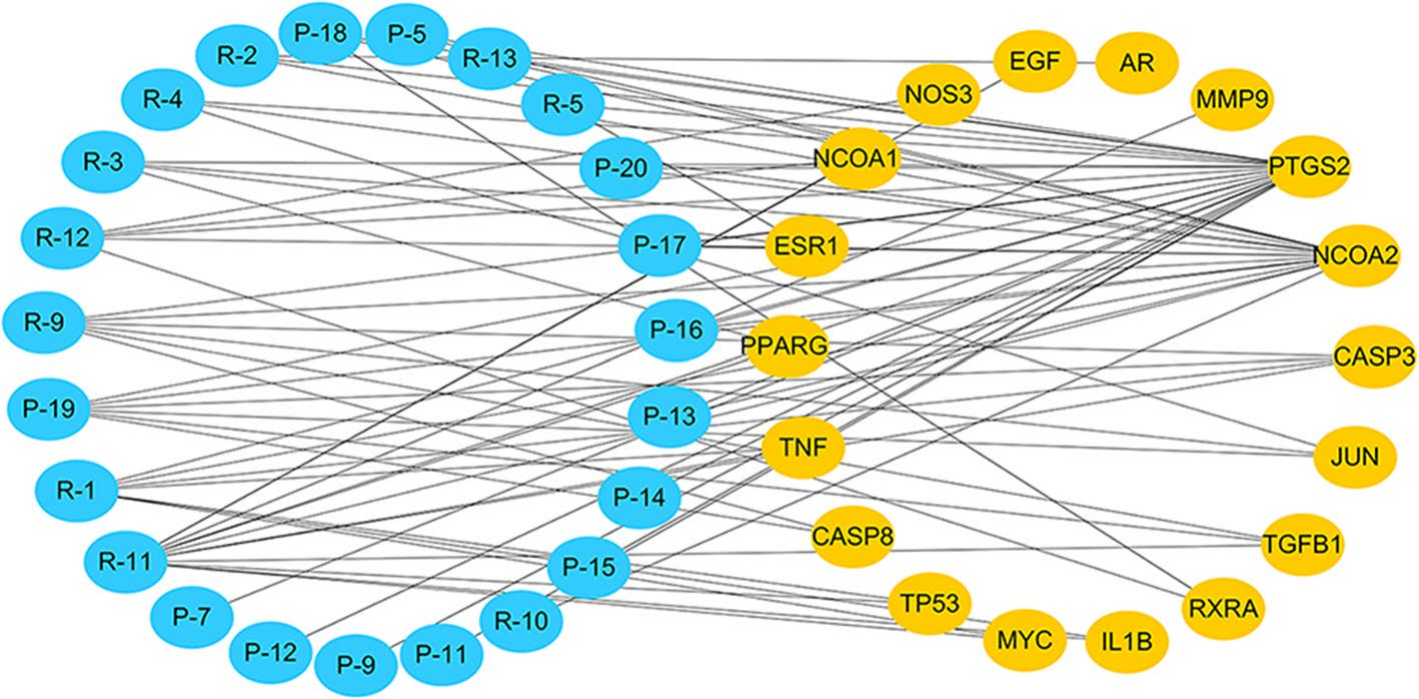
Significant hub node-Target Network of RP-active compounds-EMs targets. The orange nodes represent the influential hub nodes. The blue nodes represent the compounds (arranged by degree value)

The 18 key targets obtained by topological analysis correspond to 23 active components, and an "ingredient-target" network diagram was established to analyze the relationship between them, as shown in Fig. 5. Rhein, aloe-emodin and β-sitosterol were correlated with 12, 7 and 6 key targets respectively, ranking the top three. The results showed that RP may have the characteristics of multi-component and multi-target in treating EMs.

#### “Modules -target-pathway” network construction

**Fig. 6:**
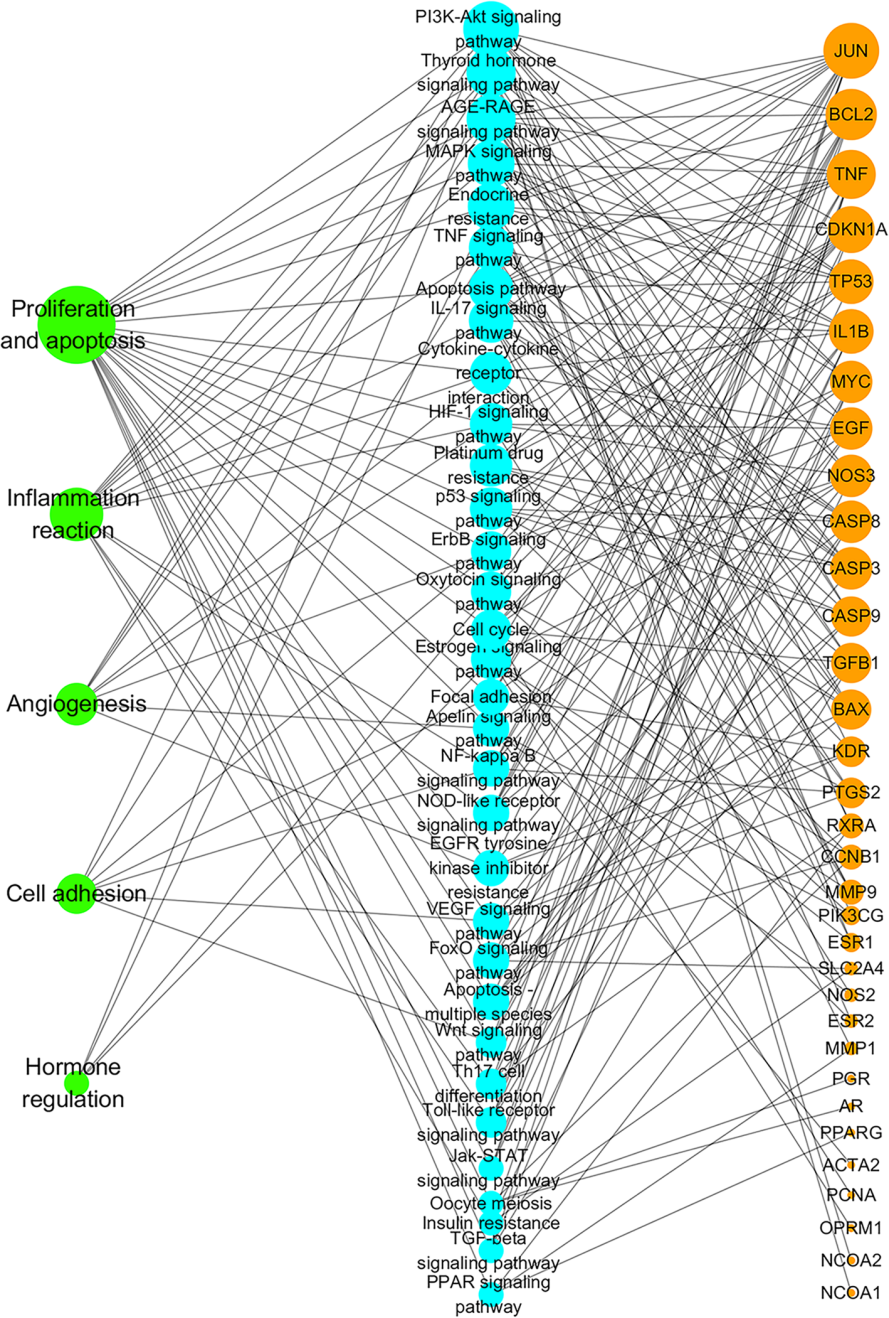
Target-pathway network of RP treating EMs. The blue nodes represent pathways, the orange nodes represent targets, and the green nodes represent the pathways' regulatory modules. The size of the node represents the value of the degree

A “module-target-pathway” network that includes 70 nodes (5 modules, 33 targets, and 32 paths) was constructed. As shown in Fig. 6, proliferation and apoptosis were presumed to be the most important mechanisms due to the high proportion of signaling pathways (24/32) and target proteins (32/33) involved.

### Test and verify

#### Selection of disease apoptotic targets for molecular docking and in vitro verification

**Fig. 7:**
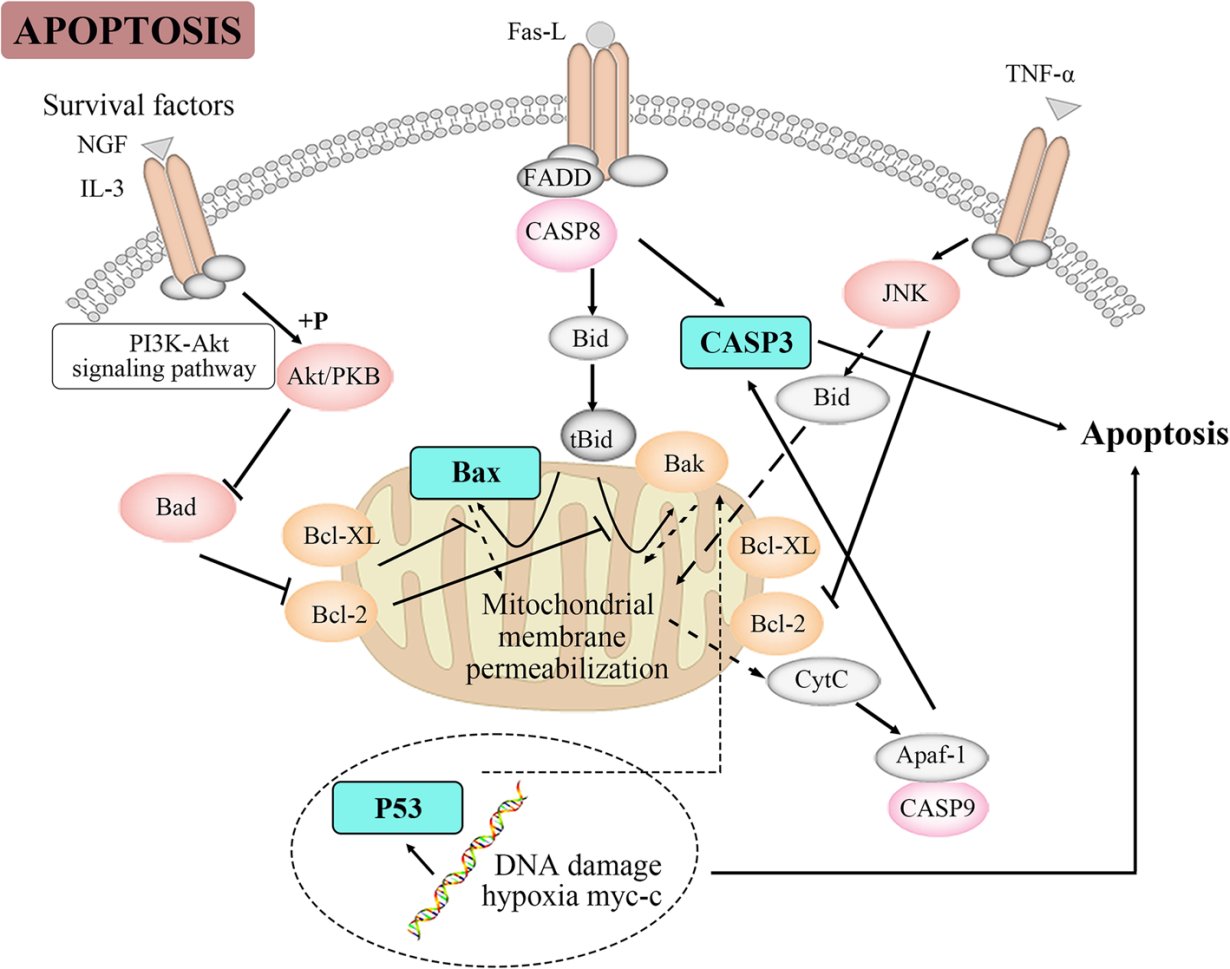
Pathway map of RP prescription against EMs. Blue indicates some of the targets selected by network pharmacology, which play a central role in the regulatory pathway of apoptosis

After identifying apoptosis regulation (hsa04210) of RP as the essential regulation modules in treating EMs, we selected P53, BAX, and CASP3 as potential therapeutic targets [29]. This was based on integrating the results of network pharmacological screening with knowledge of apoptosis regulatory signaling pathways (as shown in Fig. 7).

#### Selection of the ingredients in RP for molecular docking

Our topological analysis has successfully identified 22 ingredients out of the total 32 active ingredients in RP, which corresponded to 18 key targets, as described in article 3.5.1. It is noteworthy that 72.73% (16/22) of these active ingredients were associated with apoptosis. We conducted a comprehensive review of literature and identified 9 components that lacked significant pharmacological effects in humans. Subsequently, we shortlisted the remaining 13 components for molecular docking verification, which are aloe-emodin, beta-sitosterol, hederagenin, emodin, eupatin, physcion, rhein, crysophanol, crysophanol, (-)-catechin, toralactone, daucosterol_qt, and amygdalin.

#### Molecular docking

**Fig. 8:**
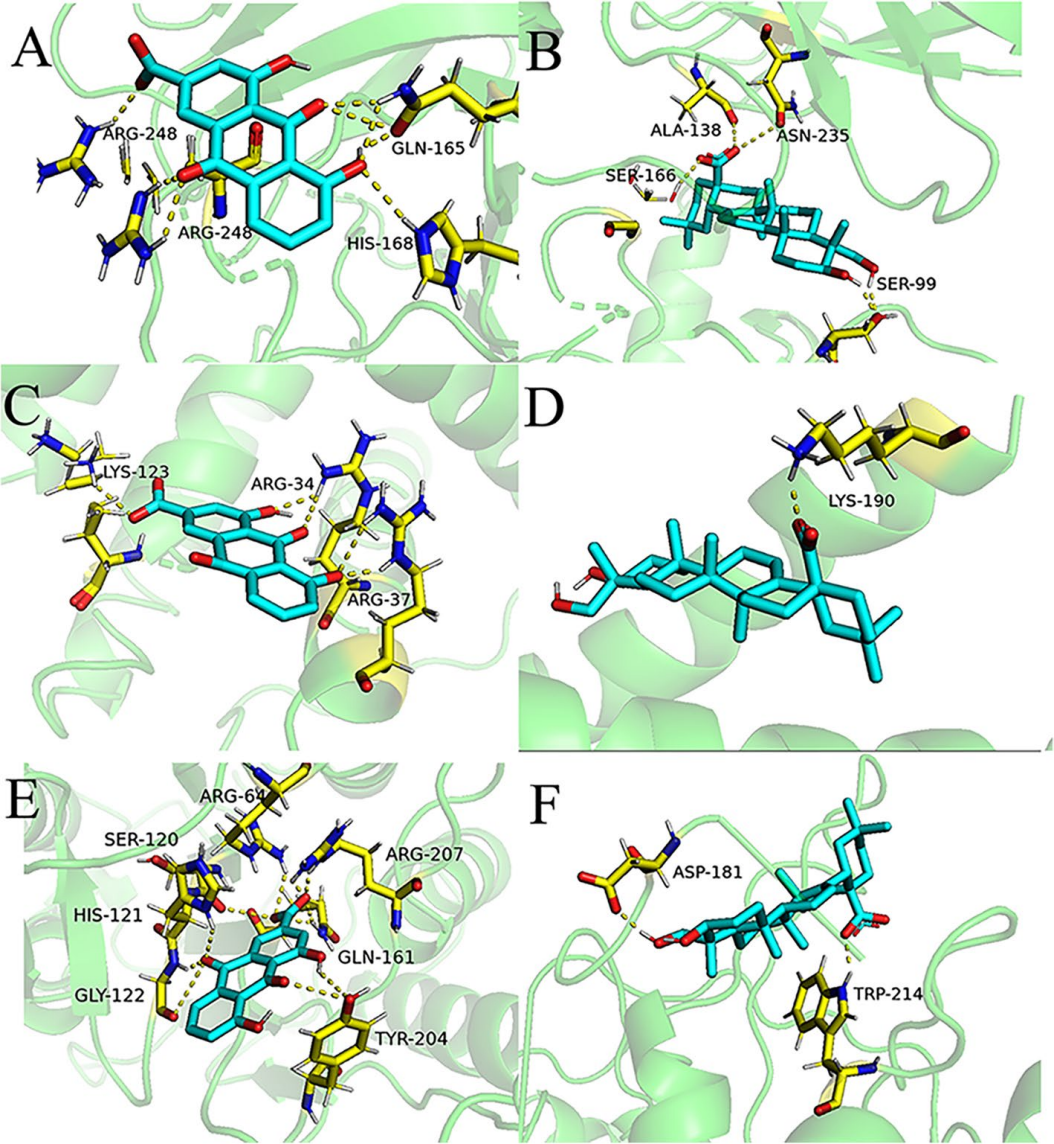
Molecular docking patterns showed that the ingredients can bind to proteins stably. **A, C, **and** E** The combination of rhein to TP53(6SHZ), BAX(6E6B) and CASP3(1NME); **B, D, **and** F** The combination of hegeragenin to TP53(6SHZ), BAX(6L95) and CASP3(3H0E); Blue amino acid residues represent the ingredient, yellow represents protein

In order to ensure the reliability of docking results, each target was docked with two different PDB IDs. The binding energy of the ingredients in RP and the protein target was shown in Table 2. With a binding energy of less than -5 kcal/mol, it can be inferred that the ingredients have a strong ability to bind with the protein target. The 3D binding patterns of partly ligands and receptors are shown in Fig. 8.

**Table 2:** The binding energy between the essential active components and the main pro-apoptotic targets

Protein Target	PDB ID	Ingredients	Binding Energy (Kcal/ Mol)	Average Binding Energy
TP53	6SHZ,6SI3	beta-sitosterol	-6.71, -8.08	-7.395
		physcion	-7.21, -7.51	-7.36
		crysophanol	-7.25, -7.53	-7.39
		aloe emodin	-7.0, -6.54	-6.77
		rhein	-7.83, -6.64	-7.235
		emodin	-7.04, -7.06	-7.05
		eupatin	-6.33, -5.54	-5.935
		hederagenin	-9.09, -8.48	-8.785
		campesterol	-7.58, -7.44	-7.51
		daucosterol_qt	-9.39, -9.47	-9.43
		toralactone	-7.36, -8.12	-7.74
		(-)-catechin	-7.8, -8.85	-8.325
		amygdalin	-4.75, -4.94	-4.845
BAX	6EB6, 6L95	beta-sitosterol	-8.04, -6.87	-7.455
		physcion	-7.95, -5.38	-6.665
		crysophanol	-5.61, -5.43	-5.52
		aloe emodin	-5.7, -5.03	-5.365
		rhein	-6.49, -5.73	-6.11
		emodin	-5.9, -5.48	-5.69
		eupatin	-7.74, -4.81	-6.275
		hederagenin	-6.37, -6.86	-6.615
		campesterol	-6.01, -6.0	-6.005
		daucosterol_qt	-5.86, -5.38	-5.62
		toralactone	-5.93, -4.15	-5.04
		(-)-catechin	-4.89, -4.58	-4.735
		amygdalin	-2.79, -1.88	-2.335
CASP3	3H0E,1NME	beta-sitosterol	-8.67, -8.33	-8.5
		physcion	-6.49, -6.56	-6.525
		crysophanol	-6.43, -5.95	-6.19
		aloe emodin	-7.03, -6.38	-6.705
		rhein	-6.31, -8.22	-7.265
		emodin	-6.28, -7.21	-6.745
		eupatin	-5.5, -6.45	-5.975
		hederagenin	-7.85, -6.81	-7.33
		campesterol	-9.13, -8.32	-8.725
		daucosterol_qt	-7.3, -6.58	-6.94
		toralactone	-6.37, -6.76	-6.565
		(-)-catechin	-5.6, -5.96	-5.78
		amygdalin	-2.76, -3.99	-3.375

#### Immortalized human endometrial stromal cells

**Fig. 9:**
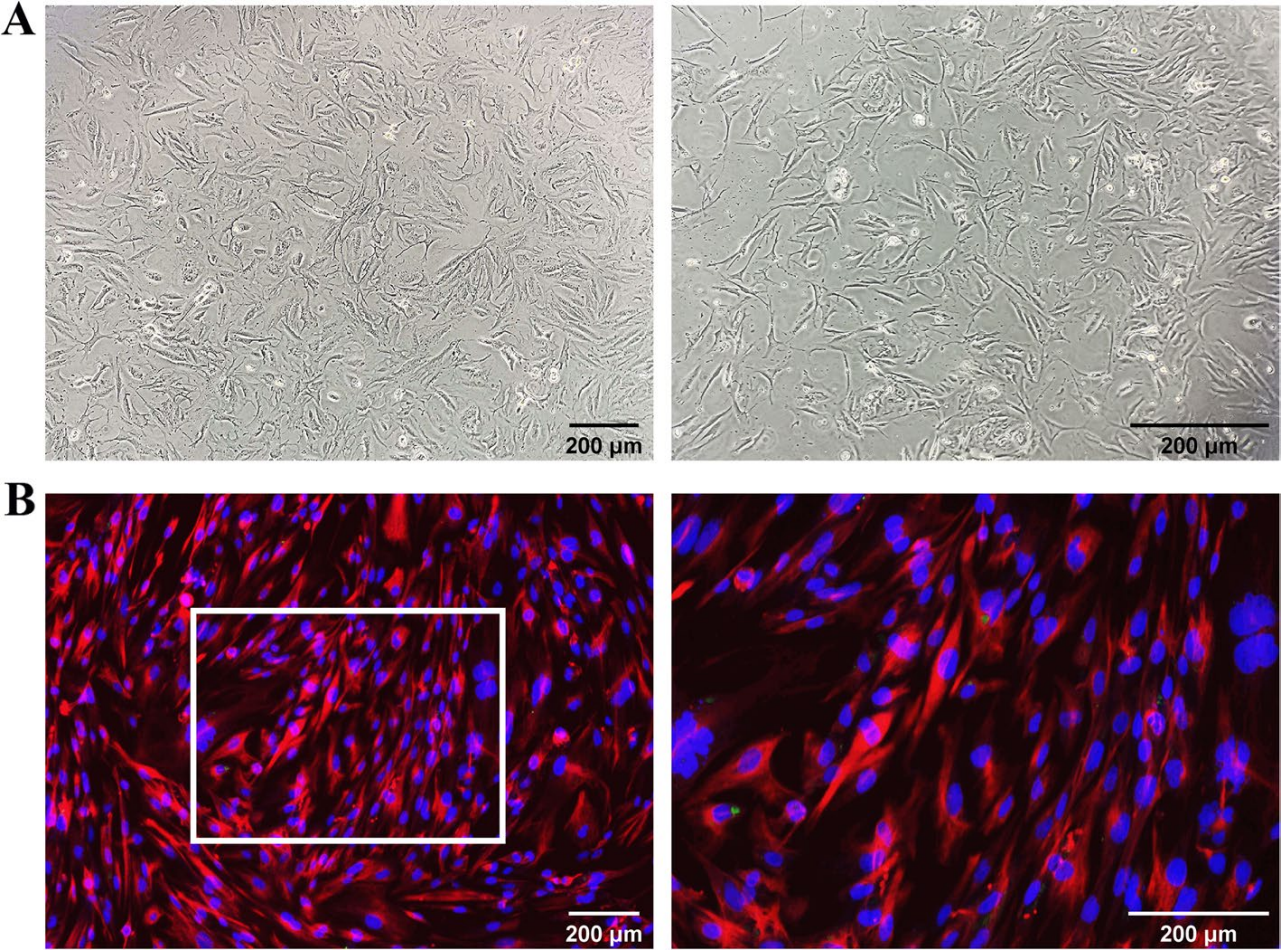
Primary human endometrial stromal cells. **A** Images of human endometrial stromal cells under optical microscope. **B** Immunofluorescence images of endometrial stromal cells. The blue represents the nucleus and the red represents the vimentin

The human endometrial stromal cells (primary cells) were spindle-shaped or star-shaped. The cytoplasm of the cells was intact, and the nuclei was large and round, with hyperplastic fibrous tissue (Fig. 9A). Vimentin of cells was detected by immunofluorescence staining, as shown in Fig. 9B.

#### Inhibition of hEM15a cell growth in vitro

**Fig. 10:**
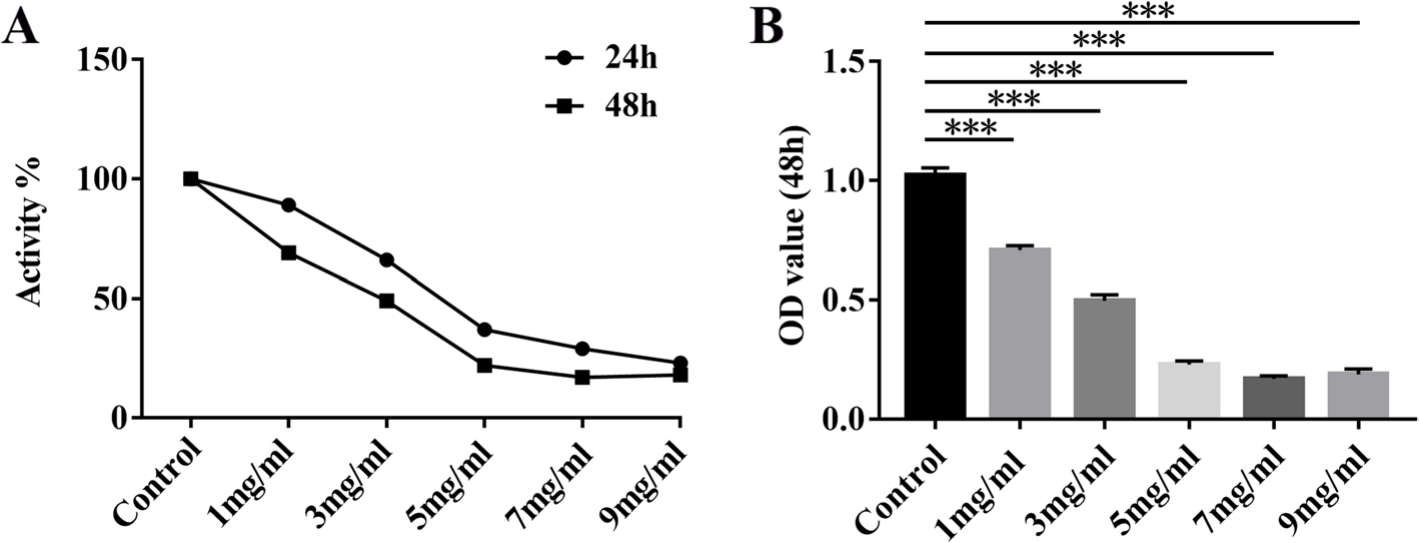
Time and dose-dependent effects of RP treatment on the viability of hEM15a cells. **A** and **B** The viability of hEM15a cells was assessed using MTT assay. ***P < 0.001 versus the control group

To verify the effect of RP on hEM15a cells, we measured the viability of hEM15a cells treated with Rhubarb and Peach kernel (ratio of 1:1) at different concentrations (1, 3, 5, 7, 9 mg/ml). In comparison with the control group, different concentrations of drugs significantly inhibited the proliferation of hEM15a cells in a time-dependent and dose-dependent manner, as shown in Fig. 10. The half-maximal inhibitory concentration (IC50) value for 48 h treatment of RP was about 3 mg/ml for hEM15a cells.

#### HEM15a cell apoptosis in vitro

**Fig. 11:**
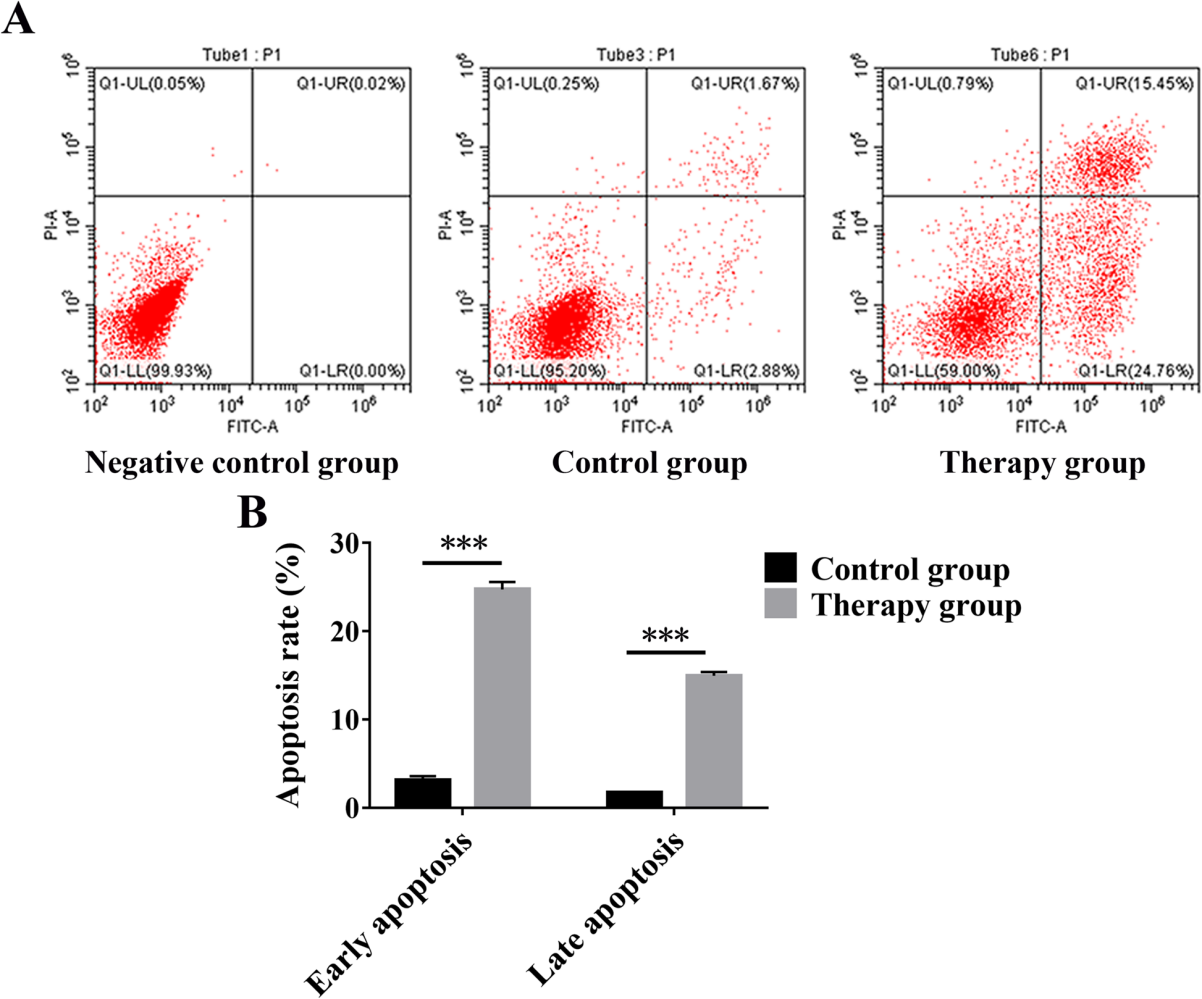
RP increased the early and late apoptosis of the hEM15a. **A** The apoptosis of hEM15a cells was detected by flow cytometry. **B** Quantitative analysis of early and late apoptosis of hEM15a cells. ***P < 0.001 versus the control group

Compared with the control group, the percentage of early and late apoptotic cells increased significantly after treatment with RP (3 mg/ml), as shown in Fig. 11.

#### Western blotting

**Fig. 12:**
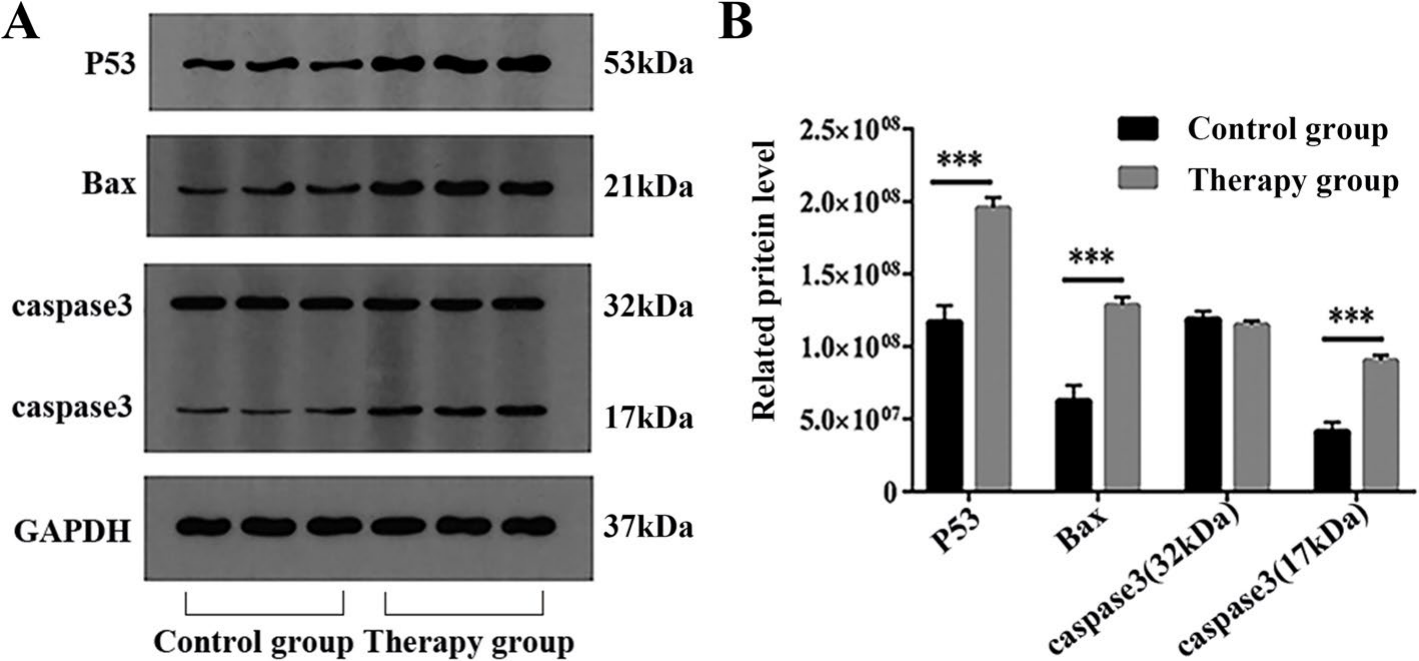
RP inhibited the protein P53, Bax, and caspase3 (17 kDa) levels of the hEM15a cell. **A** Western blotting assay analyzed the levels of P53, BAX, and CASP3 in hEM15a cells of the control group and the treatment group. **B** Quantitative analysis of the P53, Bax, caspase3 (32 kDa), and caspase3 (17 kDa) protein levels. Data were expressed as means ± SD, ***P < 0.001

Western blot analysis showed that the protein levels of P53, BAX, and CASP3 in hEM15a cells treated with RP were significantly higher than those in the control group (*P* < 0.05 or *P* < 0.01), as shown in Fig. 12.

## Discussion

The inability of current remedies to treat all aspects of EMs needs to search for effective therapies. Although RP has been reported to alleviate many symptoms of EMs [30], the active ingredients and related mechanisms that contribute to its therapeutic activity in EMs are far from clear. Considering the multi-component, multi-target of TCM, a network pharmacology method combined with molecular docking and experimental verification was here used to uncover the mechanisms of RP against EMs.

EMs is a chronic, painful disease characterized by the presence of endometrial tissue exterior to the uterus, however, the exact pathophysiology of this disease remains uncertain. The results of KEGG in our research focused on 32 pathways, primarily involving apoptosis and proliferation, inflammation reaction, angiogenesis, cell adhesion, and hormone regulation. The target proteins associated with RP pharmacological effect on EMs were retrieved, including JUN, TP53, EGF, TNF, MYC, ESR1, PPARG, PTGS2, MMP9, CASP3, IL1B, TGFB1, AR, CASP8, NOS3, NCOA2, NCOA1, and RXRA. It can be seen from the “Modules -Target-Pathway” network that 75% (24/32) of signaling pathways and almost all (32/33) target proteins are related to apoptosis and proliferation. During the progression of endometriosis, there are increased proliferation of epithelial and stromal cells as evidenced by in vitro and in vivo experiment [31]. And the resistance to apoptosis is also confirmed to play a vital role in the development and progression of EMs [6]. We speculated that proliferation and apoptosis may be the main mechanism of RP in the treatment of AM. Following that, the hEM15A cell line, sourced from the China Center for Type Culture Collection, was employed to substantiate the speculation.This specific cell line is categorized as immortalized cells and originated from eutopic endometrial stromal cells collected from patients suffering from EMs.What's notable is that these cells preserve the typical characteristics of endometrial stromal cells that are related to endometriotic disease, with a non-tumorigenic disposition and a normal diploid karyotype being evident. Notably, we demonstrated that the cell line could be propagated consistently up to a period of 5–6 passages without any discernible modifications in its morphology or function. Results of the MTT test confirmed that RP can significantly inhibit EMs cell proliferation. Additionally, flow cytometry analysis in our research revealed that treatment with RP can effectively induce early and late apoptosis in hEM15a cells.

During EMs, the survival of endometriotic cells could be due, at least in part, to the reduction of apoptotic-mediated pathways [6]. It has been demonstrated that the induction of the apoptosis led to the reduction in the EMs lesions' volume, area and diameter [32]. The result of KEGG showed that the P53 and apoptosis pathway related to promoting apoptosis might be the potential mechanisms of RP in treating EMs. P53 protein, a tetramer composed of 393 amino acids [33], has regulatory effects on apoptosis, cell cycle arrest, DNA repair, anti-oxidation, anti-angiogenesis and autophagy [34], contributing to the inhibition of the abnormal proliferation. Studies suggested that P53 played an essential role in the pathogenesis of EMs [35], and the polymorphism of the TP53 gene is related to the high risk of EMs [36]. It was reported that abnormal expressions of P53 in the ovarian cortex surrounding EMs, indicating the disorder of apoptosis in the ovarian cortex, is responsible for impaired fertility by reducing ovarian reserve and the production of good-quality oocytes [37]. BAX is a core protein in the apoptosis pathway, it can antagonize the expression of Bcl-2 and increase the pro-apoptotic gene [38]. It is evidenced that BAX can active caspase-3 via promoting the ratio of Bcl-2/Bax [39, 40], which contributes to the apoptosis of ectopic cells in EMs [41]. It has been confirmed that the ESCs in EMs exhibited a significantly lower caspase-3 gene expression compared with non-EMs [42].

The binding ability between the main components and apoptotic proteins was detected by the molecular docking technique. According to the network pharmacology, the triterpenes including beta-sitosterol, campesterol and hegeragenin are critical components of RP for treating EMs. It has been reported that beta-sitosterol, one of the triterpenes compound contained in both Rhubarb and Peach kernel, exert a promising anti-EMs effect [43, 44]. Beta-sitosterol has been confirmed in up-regulating pro-apoptotic markers P53, BAX, and CASP3 to induce apoptosis [45], which was verified by our molecular docking. Hederagenin is another triterpenoid compound contained in Peach kernel, which can activate P53 in cells and activate intrinsic apoptotic pathways through cleaved caspase-3 and Bax [46]. It has been demonstrated that hederagenin has strong binding energy with P53, BAX, and CASP3 in our research. Besides, flavonoid was detected as another key ingredient of RP in anti-EMs. a variety of flavonoids have been confirmed to induce apoptosis and inhibited proliferation in EMs cells [47–49]. The result of our molecular docking also showed eupatin, a kind of flavonoid contained in Rhubarb, had high binding energy with BAX. Moreover, Anthraquinones, including rhein, emodin, aloe-emodin, and chrysophanol, are the primary components of rhubarb, have been proven to have stable binding forces with P53, BAX, and CASP3. Previous experiments have confirmed that physcion can induce cell apoptosis by down-regulating the expression of Bcl-2, up-regulating the expression of Bax and activating the caspase-3 pathway [50]. Emodin has also been demonstrated to inhibit migration and enhance apoptotic in human ESCs [6, 51]. Similarly, studies suggested that crysophanol and Rhein can enhance the expression of P53 and Bax and induce caspase-3 activation [37, 52]. In vitro experiments, the expressions of P53, Bax, and CASP3 proteins in the RP treatment group were significantly increased compared with the control group, consistent with the prediction of network pharmacology and molecular docking. Thus, our data showed that the therapy of RP partly attribute to reduced endometriosis-induced apoptosis resistance. Further, Anthraquinones, flavonoids and triterpenoids are the possible pro-apoptotic components in RP.

## Conclusion

We identified that Anthraquinones, flavonoids and triterpenoids are considered potential active components in RP by network pharmacology, and the results of KEGG enrichment highlight the potential of RP as the target to treat EMs by inducing apoptosis, P53 signalings. Furthermore, it was confirmed that active components had high binding energy with P53, BAX, and CASP3 via molecular docking. The results of in vitro experiments detected the depressed proliferation and increased apoptosis of EMs cells in RP treated group. The levels of P53, BAX, and CASP3 significantly increased in the RP group compared with the control group. Taken together, our results suggest that RP has an anti-EMs effect by activating apoptosis, P53 signalings. Anthraquinones, flavonoids and triterpenoids might be the possible pro-apoptotic components in RP. Therefore, RP could be employed as an alternative medical approach for endometriosis treatment.

However, the computational analysis also has some limitations. The targets of the drugs and diseases we study mainly rely on existing databases and statistical analysis, so there are certain limitations. We mainly verify our analysis results through cell experiments. Therefore, other animal experiments and clinical experimental studies are still needed to verify these findings.

### Supplementary Information

**Additional file 1.**

## Data Availability

The datasets generated or analysed during the current study available from the corresponding author on reasonable request.

## Abbreviations

RP: Rhubarb-Peach Kernel
EMs: Endometriosis
TCMSP: Traditional Chinese Medicine Systems Pharmacology
BATMAN-TCM: Tool for Molecular mechanism of Traditional Chinese Medicine
STRING: Search Tool for the Retrieval of Interaction Gene/Proteins
PPI: Protein–protein interaction
KEGG: Kyoto encyclopedia of genes and genomes
OB: Oral bioavailability
DL: Drug-likeness
DC: Degree Centrality
BC: Betweenness Centrality
PDB: RCSB Protein Data Bank
