# Supplementary material for: Network pharmacology prediction and experimental verification of Rhubarb-Peach Kernel promoting apoptosis in endometriosis
Source: BMC Complement Med Ther. 2023 Aug 19;23:291. doi: 10.1186/s12906-023-04084-8 (PMC10439631; doi:10.1186/s12906-023-04084-8)
Supplement: Supplementary file 1 — Additional file 1. [file 12906_2023_4084_MOESM1_ESM.docx]

**Supplementary Material**

**Chemical Characterization Analysis of RP**

**1.Chemicals and materials**

Rhubarb granula(Approval no. 19004961) and Peach kernel granula (Approval no. 19006412) were purchased from Beijing Kangrentang Pharmaceutical Co., Ltd (Beijing, China).

Six standard substances aloe-emodin (batch No. 110795-201710), rhein (batch No. 110757-201607), emodin (batch No. 110756-201512), chrysophanol (lot No. 110796-201621), physcion (lot No. 110758-201621) were provided by the National Institutes for Food and Drug Control (Beijing, China), and the amygdalin reference substance (batch No. K-001-171216) were offered by Chengdu Ruifensi Biotechnology Co., Ltd (Chengdu, China).

**2. Preparation of** **reference substance and RP sample**

Rhubarb standard stock solutions of aloe-emodin (50 ug/ml), rhein (50 ug/ml), emodin (50 ug/ml), chrysophanol (50 ug/ml) and physcione (25 ug/ml) were prepared in methanol. 2 ml of each of the above reference solutions was prepared and mixed well (1ml of Rhubarb control solution contained aloe-emodin, rhein, emodin and chrysophanol 10 ug respectively and physcione 5ug). Amygdalin, used as quantitative stands of Peach kernel, was precisely weighted and diluted with 1ml of 70% methanol to 100ug/ml solution.

The Rhubarb formula granules were pulverized into homogenous powder, and then the powder (0.15 g) was dissolved with 25 ml of 70% methanol proceed by heating reflux for 1h. After solvent volatilization, 10 ml of 8% hydrochloric acid was subjected to mix into 5 ml of filtrate, followed by sonication for 2 min and then by trichloromethane extraction. The trichloromethane layer, sprayed as a dry residue, was reconstituted in methanol for quantitative analysis (1 ml of sample solution for every 3 mg of extract granules).

Peach kernel granula (0.1 g) was reflux-extracted 50 ml of 70% methanol by conducting ultrasonic treatment (power 250 W, frequency 50 kHz) for 30 min. After filtration, 5 ml of filtrate was taken and added to 70% methanol at a constant volume to 10 ml for HPLC analysis (1 ml of sample solution for every 1 mg of extract granules).

1. **HPLC analysis**

Series LC-2010A Liquid Chromatograph instrument (Shimadzu, Japan) was employed to analyze the extract solution and the chromatographic separation Rhubarb and Peach kernel Sample was per-formed using Dikmate (No. 20190125C18) and Hyrsil (No. 20190224C18) held at 20 ℃ - 25 ℃ with the injection volume of 10 μL, respectively. Rhubarb and Peach kernel granula were individually was eluted with the mobile phase of methanol-0.1% phosphoric acid solution (85:15) and methanol-water (1:4). The retention time and area of the reference substance were investigated through the reference substance test, and the chromatographic peaks and component content of RP in HPLC fingerprint were identified respectively. Each Gram of RP extract granules contained no less than 7.5 mg of aloe-emodin, rhein, emodin, chrysophanol, physcion and no less than 40 mg of amygdalin, which were met formulated granules standard requirement of the China National Medical Products Administration standard for Rhubarb (YBZ-PFKL-2021075) and Peach kernel (YBZ-PFKL-2021120).

Table S1 Retention time and Area (microvolts * second) of the six compounds of RP reference substance

| **compound** | **retention time(min)** | **Area (microvolts *second)** | **Herb source** |
| --- | --- | --- | --- |
| **aloe-emodin** | 6.169 | 8143.550 | Rhubarb |
| **rhein** | 7.704 | 7467.349 | Rhubarb |
| **emodin** | 12.190 | 9865.992 | Rhubarb |
| **chrysophanol** | 16.820 | 12112.638 | Rhubarb |
| **physcione** | 23.499 | 11833.638 | Rhubarb |
| **amygdalin** | 10.513 | 1029171 | Peach kernel |

Table S2 Retention time and Area (microvolts * second) of the six compounds of RP sample

| **compound** | **retention time(min)** | **Area (microvolts * second)** | **Herb source** |
| --- | --- | --- | --- |
| **aloe-emodin** | 6.179 | 8711.313 | Rhubarb |
| **rhein** | 7.728 | 7538.207 | Rhubarb |
| **emodin** | 12.228 | 9761.478 | Rhubarb |
| **chrysophanol** | 16.858 | 12187.684 | Rhubarb |
| **physcione** | 23.544 | 11816.460 | Rhubarb |
| **amygdalin** | 10.592 | 851472 | Peach kernel |


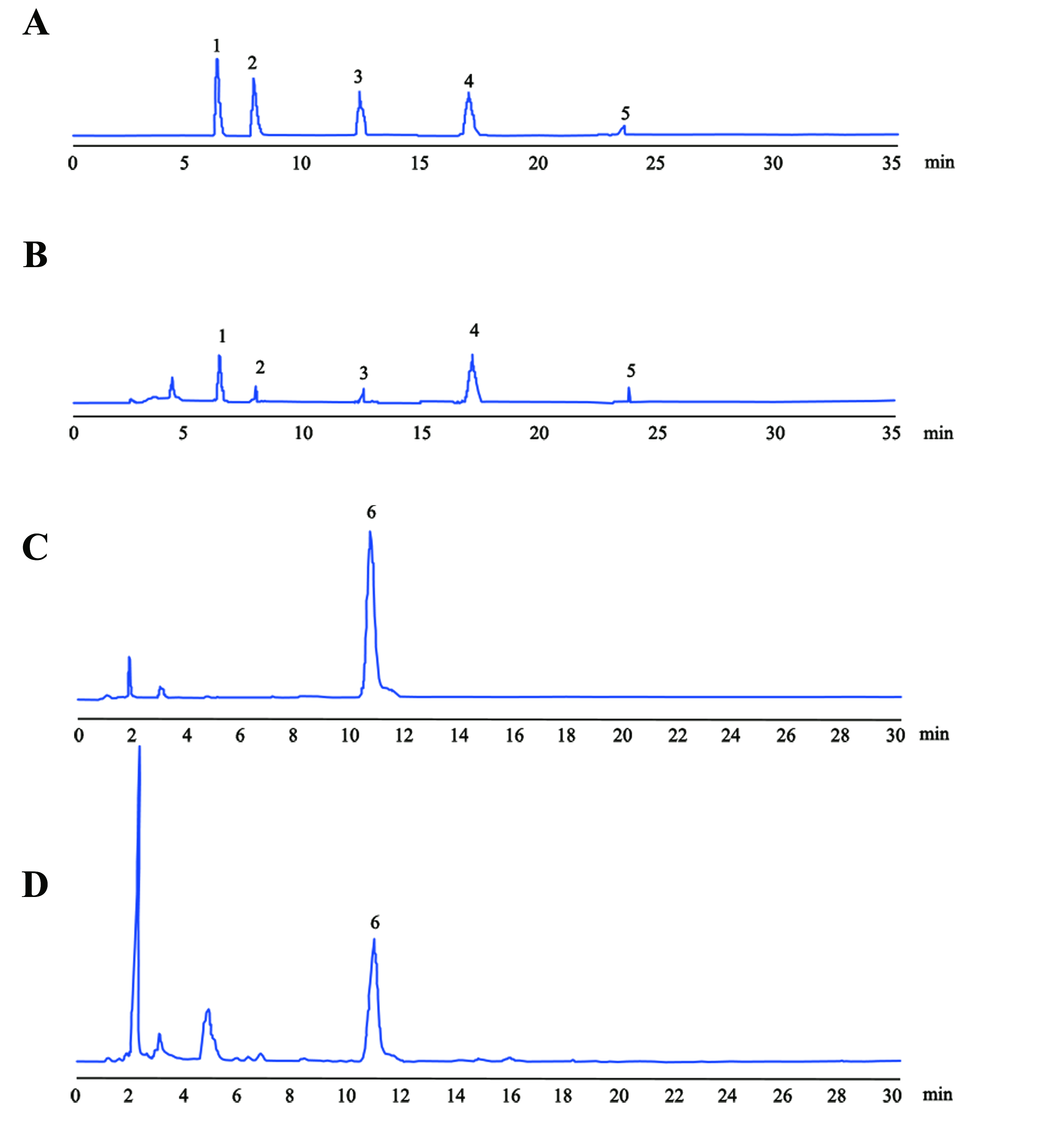


Figure S1 HPLC chromatograms fingerprint analysis of RP granula. (A) Five reference standard compounds (detected under the switched multi: wavelength). (B) Peaks 1-5 of Rhubarb sample (deteted under the switched multi-wavelength): aloe-emodin (3.33 mg/g), rhein (3.33mg/g), emodin (3.33 mg/g), chrysophanol (3.33 mg/g), physcione (1.67mg/g). (C) Peach kernel reference standard substance (detected at 210 nm wavelength). (D) Peak 6 (detected at 403 nm wavelength): amygdalin(100mg/g).

Table S3:Ingredients in RP before screening

| **Herb** | **Mol ID** | **Molecule Name** | **OB (%)** | **DL** |
| --- | --- | --- | --- | --- |
| Rhubarb | MOL001237 | [o-Acetyltoluene](https://old.tcmsp-e.com/molecule.php?qn=1237) | 38.96 | 0.02 |
|  | MOL001301 | [cis-Zimtsaeure](https://old.tcmsp-e.com/molecule.php?qn=1301) | 38.19 | 0.03 |
|  | MOL001456 | [citric acid](https://old.tcmsp-e.com/molecule.php?qn=1456) | 56.22 | 0.05 |
|  | MOL001729 | [Crysophanol](https://old.tcmsp-e.com/molecule.php?qn=1729) | 18.64 | 0.21 |
|  | MOL001794 | [MAE](https://old.tcmsp-e.com/molecule.php?qn=1794) | 65.06 | 0.01 |
|  | MOL001880 | [OXL](https://old.tcmsp-e.com/molecule.php?qn=1880) | 29.68 | 0.01 |
|  | MOL001986 | [β-sitosterol](https://old.tcmsp-e.com/molecule.php?qn=1986) | 5.84 | 0.71 |
|  | MOL002230 | [(+)-Catechin-pentaacetate](https://old.tcmsp-e.com/molecule.php?qn=2230) | 27.58 | 0.77 |
|  | MOL002231 | [(-)-Epicatechin-pentaacetate](https://old.tcmsp-e.com/molecule.php?qn=2231) | 8 | 0.77 |
|  | MOL002232 | [2-Cinnamoyl-glucose](https://old.tcmsp-e.com/molecule.php?qn=2232) | 17.02 | 0.22 |
|  | MOL002233 | [2-Methyl cardol](https://old.tcmsp-e.com/molecule.php?qn=2233) | 3.56 | 0.28 |
|  | MOL002234 | [2-Methyl-5-carboxymethyl-7-hydroxychromanone](https://old.tcmsp-e.com/molecule.php?qn=2234) | 14.8 | 0.12 |
|  | MOL002235 | [EUPATIN](https://old.tcmsp-e.com/molecule.php?qn=2235) | 50.8 | 0.41 |
|  | MOL002236 | [3,5-Di-O-galloyl-4-O-digalloylquinic acid](https://old.tcmsp-e.com/molecule.php?qn=2236) | 3.01 | 0.31 |
|  | MOL002237 | [3-Carboxy-4-hydroxy-phenoxy glucoside](https://old.tcmsp-e.com/molecule.php?qn=2237) | 13.85 | 0.22 |
|  | MOL002238 | [3-Hydroxy-25-norfriedel-3,1(10)-dien-2-one-30-oic acid](https://old.tcmsp-e.com/molecule.php?qn=2238) | 18.4 | 0.78 |
|  | MOL002239 | [5-acetyl-7-hydroxy-2-methyl-chromone](https://old.tcmsp-e.com/molecule.php?qn=2239) | 30.25 | 0.1 |
|  | MOL002240 | [5-Carboxy-7-hydroxy-2-methyl-benzopyran-gamma-one](https://old.tcmsp-e.com/molecule.php?qn=2240) | 34.4 | 0.11 |
|  | MOL002241 | [Aloeemodin-omega-O-beta-D-glucopyranoside](https://old.tcmsp-e.com/molecule.php?qn=2241) | 9.04 | 0.81 |
|  | MOL002242 | [TPBO](https://old.tcmsp-e.com/molecule.php?qn=2242) | 24.23 | 0.03 |
|  | MOL002243 | [Anthraglycoside B](https://old.tcmsp-e.com/molecule.php?qn=2243) | 27.06 | 0.8 |
|  | MOL002244 | [Chrysophanol glucoside](https://old.tcmsp-e.com/molecule.php?qn=2244) | 20.06 | 0.76 |
|  | MOL002245 | [Chrysophanol-8-O-beta-D-(6'-O-galloyl)-glucopyranoside](https://old.tcmsp-e.com/molecule.php?qn=2245) | 1.92 | 0.69 |
|  | MOL003353 | [Emodinanthrone](https://old.tcmsp-e.com/molecule.php?qn=3353) | 24.72 | 0.21 |
|  | MOL002247 | [Emodin-6-glucoside](https://old.tcmsp-e.com/molecule.php?qn=2247) | 16.09 | 0.8 |
|  | MOL002248 | [Gallic acid-4-O-(6'-O-galloyl)-glucoside](https://old.tcmsp-e.com/molecule.php?qn=2248) | 27.06 | 0.67 |
|  | MOL002249 | [gallocatechin](https://old.tcmsp-e.com/molecule.php?qn=2249) | 2.26 | 0.27 |
|  | MOL002250 | [[(2R,3R,4S,5R,6R)-3,5-dihydroxy-2-(3,4,5-trihydroxybenzoyl)oxy-6-[(3,4,5-trihydroxybenzoyl)oxymethyl]oxan-4-yl] 3,4,5-trihydroxybenzoate](https://old.tcmsp-e.com/molecule.php?qn=2250) | 3.01 | 0.54 |
|  | MOL002251 | [Mutatochrome](https://old.tcmsp-e.com/molecule.php?qn=2251) | 48.64 | 0.61 |
|  | MOL002252 | [palmidin B](https://old.tcmsp-e.com/molecule.php?qn=2252) | 1.21 | 0.69 |
|  | MOL002253 | [Palmidin C](https://old.tcmsp-e.com/molecule.php?qn=2253) | 2.35 | 0.69 |
|  | MOL002254 | [Barolub](https://old.tcmsp-e.com/molecule.php?qn=2254) | 16.29 | 0.22 |
|  | MOL002366 | [Rheochrysin](https://old.tcmsp-e.com/molecule.php?qn=2366) | 18.31 | 0.82 |
|  | MOL002256 | [1,8-dihydroxy-3-methoxy-2,6-dimethyl-9,10-anthraquinone](https://old.tcmsp-e.com/molecule.php?qn=2256) | 5.53 | 0.29 |
|  | MOL002257 | [Physcion-8-O-beta-D-glucopyranoside](https://old.tcmsp-e.com/molecule.php?qn=2257) | 8.2 | 0.85 |
|  | MOL002258 | [Physcion-9-O-beta-D-glucopyranoside_qt](https://old.tcmsp-e.com/molecule.php?qn=2258) | 20.3 | 0.3 |
|  | MOL002259 | [Physciondiglucoside](https://old.tcmsp-e.com/molecule.php?qn=2259) | 41.65 | 0.63 |
|  | MOL002260 | [Procyanidin B-5,3'-O-gallate](https://old.tcmsp-e.com/molecule.php?qn=2260) | 31.99 | 0.32 |
|  | MOL002261 | [ZINC04081604](https://old.tcmsp-e.com/molecule.php?qn=2261) | 15.23 | 0.44 |
|  | MOL002262 | [5-[(Z)-2-(3-hydroxy-4-methoxy-phenyl)vinyl]resorcinol](https://old.tcmsp-e.com/molecule.php?qn=2262) | 76.25 | 0.15 |
|  | MOL002263 | [1-Cinnamoyl-3-hydroxy-11-methoxymeliacarpinin](https://old.tcmsp-e.com/molecule.php?qn=2263) | 35.72 | 0.07 |
|  | MOL002264 | [Rhapontisterone](https://old.tcmsp-e.com/molecule.php?qn=2264) | 4.39 | 0.82 |
|  | MOL002265 | [Rheidin B](https://old.tcmsp-e.com/molecule.php?qn=2265) | 1.21 | 0.65 |
|  | MOL002266 | [Rheidin C](https://old.tcmsp-e.com/molecule.php?qn=2266) | 1.24 | 0.58 |
|  | MOL002267 | [Rhein diglucoside](https://old.tcmsp-e.com/molecule.php?qn=2267) | 2.93 | 0.63 |
|  | MOL002268 | [rhein](https://old.tcmsp-e.com/molecule.php?qn=2268) | 47.07 | 0.28 |
|  | MOL002269 | [Rheinoside A](https://old.tcmsp-e.com/molecule.php?qn=2269) | 0.82 | 0.68 |
|  | MOL002270 | [Rheinoside A_qt](https://old.tcmsp-e.com/molecule.php?qn=2270) | 26.28 | 0.75 |
|  | MOL002271 | [Rheumin](https://old.tcmsp-e.com/molecule.php?qn=2271) | 9.16 | 0.17 |
|  | MOL002272 | [Sennidin C](https://old.tcmsp-e.com/molecule.php?qn=2272) | 1.28 | 0.61 |
|  | MOL002368 | [Sennoside A](https://old.tcmsp-e.com/molecule.php?qn=2368) | 3.34 | 0.08 |
|  | MOL002274 | [(9S)-9-[(9R)-2-carboxy-4,5-dihydroxy-10-oxo-9H-anthracen-9-yl]-4,5-dihydroxy-10-oxo-9H-anthracene-2-carboxylic acid](https://old.tcmsp-e.com/molecule.php?qn=2274) | 27.75 | 0.57 |
|  | MOL002275 | [Sennoside C](https://old.tcmsp-e.com/molecule.php?qn=2275) | 3.99 | 0.09 |
|  | MOL002276 | [Sennoside E_qt](https://old.tcmsp-e.com/molecule.php?qn=2276) | 50.69 | 0.61 |
|  | MOL006832 | [beta-Glucogallin](https://old.tcmsp-e.com/molecule.php?qn=6832) | 17.89 | 0.25 |
|  | MOL002278 | [Sennoside E](https://old.tcmsp-e.com/molecule.php?qn=2278) | 3.02 | 0.06 |
|  | MOL002279 | [Serotonin](https://old.tcmsp-e.com/molecule.php?qn=2279) | 42.99 | 0.06 |
|  | MOL002280 | [Torachrysone-8-O-beta-D-(6'-oxayl)-glucoside](https://old.tcmsp-e.com/molecule.php?qn=2280) | 43.02 | 0.74 |
|  | MOL002281 | [Toralactone](https://old.tcmsp-e.com/molecule.php?qn=2281) | 46.46 | 0.24 |
|  | MOL002282 | [[Epicatechin-(48)]5-epicatechin](https://old.tcmsp-e.com/molecule.php?qn=2282) | 4.02 | 0 |
|  | MOL002283 | [[(2R,3S,4S,5R,6S)-6-[4-[(Z)-2-(3,5-dihydroxyphenyl)ethenyl]phenoxy]-3,4,5-trihydroxyoxan-2-yl]methyl 3,4,5-trihydroxybenzoate](https://old.tcmsp-e.com/molecule.php?qn=2283) | 2.69 | 0.73 |
|  | MOL002284 | [PIT](https://old.tcmsp-e.com/molecule.php?qn=2284) | 72.29 | 0.13 |
|  | MOL002285 | [1-O-Galloyl-glycerol](https://old.tcmsp-e.com/molecule.php?qn=2285) | 63.21 | 0.1 |
|  | MOL002286 | [laccaic acid D](https://old.tcmsp-e.com/molecule.php?qn=2286) | 2.06 | 0.31 |
|  | MOL002287 | [Rheosmin](https://old.tcmsp-e.com/molecule.php?qn=2287) | 26.79 | 0.04 |
|  | MOL002288 | [Emodin-1-O-beta-D-glucopyranoside](https://old.tcmsp-e.com/molecule.php?qn=2288) | 44.81 | 0.8 |
|  | MOL002289 | [physcione](https://old.tcmsp-e.com/molecule.php?qn=2289) | 19.38 | 0.27 |
|  | MOL002290 | [Sennoside B](https://old.tcmsp-e.com/molecule.php?qn=2290) | 3.34 | 0.08 |
|  | MOL002291 | [1-O-Galloylpedunculagin](https://old.tcmsp-e.com/molecule.php?qn=2291) | 38.09 | 0.04 |
|  | MOL002292 | [Sennoside D](https://old.tcmsp-e.com/molecule.php?qn=2292) | 3.99 | 0.09 |
|  | MOL002293 | [Sennoside D_qt](https://old.tcmsp-e.com/molecule.php?qn=2293) | 61.06 | 0.61 |
|  | MOL002294 | [strumaroside](https://old.tcmsp-e.com/molecule.php?qn=2294) | 20.78 | 0.67 |
|  | MOL002295 | [cinnamic acid](https://old.tcmsp-e.com/molecule.php?qn=2295) | 19.68 | 0.03 |
|  | MOL002296 | [Daucosterol](https://old.tcmsp-e.com/molecule.php?qn=2296) | 20.18 | 0.69 |
|  | MOL002297 | [Daucosterol_qt](https://old.tcmsp-e.com/molecule.php?qn=2297) | 35.89 | 0.7 |
|  | MOL002298 | [aloeemodin](https://old.tcmsp-e.com/molecule.php?qn=2298) | 20.65 | 0.24 |
|  | MOL002299 | [DMR](https://old.tcmsp-e.com/molecule.php?qn=2299) | 68.62 | 0.02 |
|  | MOL002300 | [10beta-Hydroxy-6beta-isobutyrylfuranoeremophilane](https://old.tcmsp-e.com/molecule.php?qn=2300) | 16.94 | 0.29 |
|  | MOL002301 | [DLA](https://old.tcmsp-e.com/molecule.php?qn=2301) | 44.51 | 0.01 |
|  | MOL002302 | [RHAPONTIN](https://old.tcmsp-e.com/molecule.php?qn=2302) | 9 | 0.55 |
|  | MOL002303 | [palmidin A](https://old.tcmsp-e.com/molecule.php?qn=2303) | 32.45 | 0.65 |
|  | MOL002304 | [Glycerite](https://old.tcmsp-e.com/molecule.php?qn=2304) | 7.28 | 0.03 |
|  | MOL002305 | [2,3-Digalloylglucose](https://old.tcmsp-e.com/molecule.php?qn=2305) | 2.99 | 0.66 |
|  | MOL000346 | [succinic acid](https://old.tcmsp-e.com/molecule.php?qn=346) | 29.62 | 0.01 |
|  | MOL000358 | [beta-sitosterol](https://old.tcmsp-e.com/molecule.php?qn=358) | 36.91 | 0.75 |
|  | MOL000399 | [Docosanoate](https://old.tcmsp-e.com/molecule.php?qn=399) | 15.69 | 0.26 |
|  | MOL000471 | [aloe-emodin](https://old.tcmsp-e.com/molecule.php?qn=471) | 83.38 | 0.24 |
|  | MOL000472 | [emodin](https://old.tcmsp-e.com/molecule.php?qn=472) | 24.4 | 0.24 |
|  | MOL000476 | [Physcion](https://old.tcmsp-e.com/molecule.php?qn=476) | 22.29 | 0.27 |
|  | MOL000513 | [3,4,5-trihydroxybenzoic acid](https://old.tcmsp-e.com/molecule.php?qn=513) | 31.69 | 0.04 |
|  | MOL000554 | [gallic acid-3-O-(6'-O-galloyl)-glucoside](https://old.tcmsp-e.com/molecule.php?qn=554) | 30.25 | 0.67 |
|  | MOL000096 | [(-)-catechin](https://old.tcmsp-e.com/molecule.php?qn=96) | 49.68 | 0.24 |
| Peach kernel | MOL000131 | [EIC](https://old.tcmsp-e.com/molecule.php?qn=131) | 41.9 | 0.14 |
|  | MOL001315 | [campesterol-3-O-β-D-glucopyranoside](https://old.tcmsp-e.com/molecule.php?qn=1315) | 20.49 | 0.67 |
|  | MOL001316 | [campesterol-3-O-β-D-glucopyranoside_qt](https://old.tcmsp-e.com/molecule.php?qn=1316) | 7.86 | 0.72 |
|  | MOL001317 | [β-sitosterol 3-O-β-D-(6-O-oleyl)glucopyranoside](https://old.tcmsp-e.com/molecule.php?qn=1317) | 26.94 | 0.16 |
|  | MOL001318 | [β-sitosterol-3-(6-palmitoyl)glucopyranoside](https://old.tcmsp-e.com/molecule.php?qn=1318) | 26.07 | 0.18 |
|  | MOL001319 | [3-feruloylquinic acid](https://old.tcmsp-e.com/molecule.php?qn=1319) | 19.31 | 0.36 |
|  | MOL001320 | [Amygdalin](https://old.tcmsp-e.com/molecule.php?qn=1320) | 4.42 | 0.61 |
|  | MOL001321 | [d-mandelonitrile](https://old.tcmsp-e.com/molecule.php?qn=1321) | 48.26 | 0.02 |
|  | MOL001955 | [Heriguard](https://old.tcmsp-e.com/molecule.php?qn=1955) | 11.93 | 0.33 |
|  | MOL001323 | [Sitosterol alpha1](https://old.tcmsp-e.com/molecule.php?qn=1323) | 43.28 | 0.78 |
|  | MOL001324 | [campesterol-3-O-β-D-(6-O-oleyl)glucopyranoside](https://old.tcmsp-e.com/molecule.php?qn=1324) | 27.03 | 0.17 |
|  | MOL001325 | [campesterol-3-O-β-D-(6-O-palmityl)glucopyranoside](https://old.tcmsp-e.com/molecule.php?qn=1325) | 25.65 | 0.19 |
|  | MOL001327 | [2,3-didehydro GA69](https://old.tcmsp-e.com/molecule.php?qn=1327) | 14.28 | 0.5 |
|  | MOL001328 | [2,3-didehydro GA70](https://old.tcmsp-e.com/molecule.php?qn=1328) | 63.29 | 0.5 |
|  | MOL001329 | [2,3-didehydro GA77](https://old.tcmsp-e.com/molecule.php?qn=1329) | 88.08 | 0.53 |
|  | MOL001330 | [2,3-didehydro GA9](https://old.tcmsp-e.com/molecule.php?qn=1330) | 17.03 | 0.45 |
|  | MOL001331 | [Amygdalinic acid](https://old.tcmsp-e.com/molecule.php?qn=1331) | 4.15 | 0.63 |
|  | MOL001332 | [RMN](https://old.tcmsp-e.com/molecule.php?qn=1332) | 43.67 | 0.03 |
|  | MOL001333 | [7-dehydroavenasterol](https://old.tcmsp-e.com/molecule.php?qn=1333) | 10.03 | 0.76 |
|  | MOL001334 | [Benzyl Beta -gentiobioside](https://old.tcmsp-e.com/molecule.php?qn=1334) | 3.46 | 0.56 |
|  | MOL001335 | [WLN: Q1R](https://old.tcmsp-e.com/molecule.php?qn=1335) | 58.68 | 0.01 |
|  | MOL001336 | [(2S,3R,4S,5S,6R)-2-(benzyloxy)-6-methylol-tetrahydropyran-3,4,5-triol](https://old.tcmsp-e.com/molecule.php?qn=1336) | 17.14 | 0.14 |
|  | MOL001337 | [Benzyl glucopyranoside](https://old.tcmsp-e.com/molecule.php?qn=1337) | 12.39 | 0.14 |
|  | MOL001338 | [GA118](https://old.tcmsp-e.com/molecule.php?qn=1338) | 10.41 | 0.53 |
|  | MOL001339 | [GA119](https://old.tcmsp-e.com/molecule.php?qn=1339) | 76.36 | 0.49 |
|  | MOL001340 | [GA120](https://old.tcmsp-e.com/molecule.php?qn=1340) | 84.85 | 0.45 |
|  | MOL001341 | [GA121](https://old.tcmsp-e.com/molecule.php?qn=1341) | 14.13 | 0.5 |
|  | MOL001342 | [GA121-isolactone](https://old.tcmsp-e.com/molecule.php?qn=1342) | 72.7 | 0.54 |
|  | MOL001343 | [GA122](https://old.tcmsp-e.com/molecule.php?qn=1343) | 64.79 | 0.5 |
|  | MOL001344 | [GA122-isolactone](https://old.tcmsp-e.com/molecule.php?qn=1344) | 88.11 | 0.54 |
|  |  |  |  |  |
|  | MOL001345 | [Methyl-alpha-D-fructofuranoside](https://old.tcmsp-e.com/molecule.php?qn=1345) | 65.63 | 0.05 |
|  | MOL001346 | [GA126](https://old.tcmsp-e.com/molecule.php?qn=1346) | 11.8 | 0.53 |
|  | MOL001347 | [GA16](https://old.tcmsp-e.com/molecule.php?qn=1347) | 14.26 | 0.53 |
|  | MOL001348 | [gibberellin 17](https://old.tcmsp-e.com/molecule.php?qn=1348) | 94.64 | 0.49 |
|  | MOL001349 | [4a-formyl-7alpha-hydroxy-1-methyl-8-methylidene-4aalpha,4bbeta-gibbane-1alpha,10beta-dicarboxylic acid](https://old.tcmsp-e.com/molecule.php?qn=1349) | 88.6 | 0.46 |
|  | MOL001350 | [GA30](https://old.tcmsp-e.com/molecule.php?qn=1350) | 61.72 | 0.54 |
|  | MOL001351 | [Gibberellin A44](https://old.tcmsp-e.com/molecule.php?qn=1351) | 101.61 | 0.54 |
|  | MOL001352 | [GA54](https://old.tcmsp-e.com/molecule.php?qn=1352) | 64.21 | 0.53 |
|  | MOL001353 | [GA60](https://old.tcmsp-e.com/molecule.php?qn=1353) | 93.17 | 0.53 |
|  | MOL001354 | [GA61](https://old.tcmsp-e.com/molecule.php?qn=1354) | 14.82 | 0.49 |
|  | MOL001355 | [GA63](https://old.tcmsp-e.com/molecule.php?qn=1355) | 65.54 | 0.54 |
|  | MOL001356 | [MGL](https://old.tcmsp-e.com/molecule.php?qn=1356) | 24.46 | 0.05 |
|  | MOL001357 | [GA69](https://old.tcmsp-e.com/molecule.php?qn=1357) | 17.67 | 0.49 |
|  | MOL001358 | [gibberellin 7](https://old.tcmsp-e.com/molecule.php?qn=1358) | 73.8 | 0.5 |
|  | MOL001359 | [GA70](https://old.tcmsp-e.com/molecule.php?qn=1359) | 14.04 | 0.49 |
|  | MOL001360 | [GA77](https://old.tcmsp-e.com/molecule.php?qn=1360) | 87.89 | 0.53 |
|  | MOL001361 | [GA87](https://old.tcmsp-e.com/molecule.php?qn=1361) | 68.85 | 0.57 |
|  | MOL001362 | [GA95](https://old.tcmsp-e.com/molecule.php?qn=1362) | 20.01 | 0.49 |
|  | MOL001363 | [GA97](https://old.tcmsp-e.com/molecule.php?qn=1363) | 10.12 | 0.47 |
|  | MOL001364 | [(2S)-2-phenyl-2-[(2S,3R,4S,5S,6R)-3,4,5-trihydroxy-6-(hydroxymethyl)oxan-2-yl]oxyacetic acid](https://old.tcmsp-e.com/molecule.php?qn=1364) | 8.27 | 0.2 |
|  | MOL001365 | [prunasin](https://old.tcmsp-e.com/molecule.php?qn=1365) | 12.61 | 0.18 |
|  | MOL001366 | [MNN](https://old.tcmsp-e.com/molecule.php?qn=1366) | 48.36 | 0.02 |
|  | MOL001367 | [[(2S,3R,4S,5S,6R)-3,4,5-trihydroxy-6-(hydroxymethyl)oxan-2-yl] (E)-3-(4-hydroxyphenyl)prop-2-enoate](https://old.tcmsp-e.com/molecule.php?qn=1367) | 9.8 | 0.26 |
|  | MOL001368 | [3-O-p-coumaroylquinic acid](https://old.tcmsp-e.com/molecule.php?qn=1368) | 37.63 | 0.29 |
|  | MOL001369 | [Grandidentatin](https://old.tcmsp-e.com/molecule.php?qn=1369) | 10.56 | 0.54 |
|  | MOL001370 | [[2-[(2S,3R,4S,5S,6R)-3,4,5-trihydroxy-6-(hydroxymethyl)oxan-2-yl]oxyphenyl]methyl (E)-3-(3,4-dihydroxyphenyl)prop-2-enoate](https://old.tcmsp-e.com/molecule.php?qn=1370) | 8.22 | 0.69 |
|  | MOL001371 | [Populoside_qt](https://old.tcmsp-e.com/molecule.php?qn=1371) | 108.89 | 0.2 |
|  | MOL001372 | [beta-D-Glucopyranoside, 2-((benzoyloxy)methyl)-4-hydroxyphenyl](https://old.tcmsp-e.com/molecule.php?qn=1372) | 13.51 | 0.53 |
|  | MOL001373 | [Salireposide_qt](https://old.tcmsp-e.com/molecule.php?qn=1373) | 24.3 | 0.12 |
|  | MOL001901 | [24-Methylenecycloartanol](https://old.tcmsp-e.com/molecule.php?qn=1901) | 10.4 | 0.79 |
|  | MOL000256 | [Olein](https://old.tcmsp-e.com/molecule.php?qn=256) | 27.27 | 0.13 |
|  | MOL000295 | [alexandrin](https://old.tcmsp-e.com/molecule.php?qn=295) | 20.63 | 0.63 |
|  | MOL000296 | [hederagenin](https://old.tcmsp-e.com/molecule.php?qn=296) | 36.91 | 0.75 |
|  | MOL000358 | [beta-sitosterol](https://old.tcmsp-e.com/molecule.php?qn=358) | 36.91 | 0.75 |
|  | MOL000397 | [cis-p-Coumarate](https://old.tcmsp-e.com/molecule.php?qn=397) | 45.98 | 0.04 |
|  | MOL000493 | [campesterol](https://old.tcmsp-e.com/molecule.php?qn=493) | 37.58 | 0.71 |

Table S4:Ingredients in RP after screening

| Herb | Mol ID | Molecule Name | OB (%) | DL |
| --- | --- | --- | --- | --- |
| Rhubarb | MOL002235 | [EUPATIN](http://tcmspw.com/molecule.php?qn=2235) | 50.8 | 0.41 |
|  | MOL002251 | [Mutatochrome](http://tcmspw.com/molecule.php?qn=2251) | 48.64 | 0.61 |
|  | MOL002259 | [Physciondiglucoside](http://tcmspw.com/molecule.php?qn=2259) | 41.65 | 0.63 |
|  | MOL002260 | [Procyanidin B-5,3'-O-gallate](http://tcmspw.com/molecule.php?qn=2260) | 31.99 | 0.32 |
|  | MOL002268 | [rhein](http://tcmspw.com/molecule.php?qn=2268) | 47.07 | 0.28 |
|  | MOL002276 | [Sennoside E_qt](http://tcmspw.com/molecule.php?qn=2276) | 50.69 | 0.61 |
|  | MOL002280 | [Torachrysone-8-O-beta-D-(6'-oxayl)-glucoside](http://tcmspw.com/molecule.php?qn=2280) | 43.02 | 0.74 |
|  | MOL002281 | [Toralactone](http://tcmspw.com/molecule.php?qn=2281) | 46.46 | 0.24 |
|  | MOL002288 | [Emodin-1-O-beta-D-glucopyranoside](http://tcmspw.com/molecule.php?qn=2288) | 44.81 | 0.8 |
|  | MOL002293 | [Sennoside D_qt](http://tcmspw.com/molecule.php?qn=2293) | 61.06 | 0.61 |
|  | MOL002297 | [Daucosterol_qt](http://tcmspw.com/molecule.php?qn=2297) | 35.89 | 0.7 |
|  | MOL002303 | [palmidin A](http://tcmspw.com/molecule.php?qn=2303) | 32.45 | 0.65 |
|  | MOL000358 | [beta-sitosterol](http://tcmspw.com/molecule.php?qn=358) | 36.91 | 0.75 |
|  | MOL000471 | [aloe-emodin](http://tcmspw.com/molecule.php?qn=471) | 83.38 | 0.24 |
|  | MOL000554 | [gallic acid-3-O-(6'-O-galloyl)-glucoside](http://tcmspw.com/molecule.php?qn=554) | 30.25 | 0.67 |
|  | MOL000096 | [(-)-catechin](http://tcmspw.com/molecule.php?qn=96) | 49.68 | 0.24 |
|  | MOL000472 | [emodin](http://tcmspw.com/molecule.php?qn=472) | 24.4 | 0.24 |
|  | MOL001729 | [Crysophanol](http://tcmspw.com/molecule.php?qn=1729) | 18.64 | 0.21 |
|  | MOL000476 | [Physcion](http://tcmspw.com/molecule.php?qn=476) | 22.29 | 0.27 |
| Peach kernel | MOL001323 | [Sitosterol alpha1](http://tcmspw.com/molecule.php?qn=1323) | 43.28 | 0.78 |
|  | MOL001328 | [2,3-didehydro GA70](http://tcmspw.com/molecule.php?qn=1328) | 63.29 | 0.5 |
|  | MOL001329 | [2,3-didehydro GA77](http://tcmspw.com/molecule.php?qn=1329) | 88.08 | 0.53 |
|  | MOL001339 | [GA119](http://tcmspw.com/molecule.php?qn=1339) | 76.36 | 0.49 |
|  | MOL001340 | [GA120](http://tcmspw.com/molecule.php?qn=1340) | 84.85 | 0.45 |
|  | MOL001342 | [GA121-isolactone](http://tcmspw.com/molecule.php?qn=1342) | 72.7 | 0.54 |
|  | MOL001343 | [GA122](http://tcmspw.com/molecule.php?qn=1343) | 64.79 | 0.5 |
|  | MOL001344 | [GA122-isolactone](http://tcmspw.com/molecule.php?qn=1344) | 88.11 | 0.54 |
|  | MOL001348 | [gibberellin 17](http://tcmspw.com/molecule.php?qn=1348) | 94.64 | 0.49 |
|  | MOL001349 | [4a-formyl-7alpha-hydroxy-1-methyl-8-methylidene-4aalpha,4bbeta-gibbane-1alpha,10beta-dicarboxylic acid](http://tcmspw.com/molecule.php?qn=1349) | 88.6 | 0.46 |
|  | MOL001350 | [GA30](http://tcmspw.com/molecule.php?qn=1350) | 61.72 | 0.54 |
|  | MOL001351 | [Gibberellin A44](http://tcmspw.com/molecule.php?qn=1351) | 101.61 | 0.54 |
|  | MOL001352 | [GA54](http://tcmspw.com/molecule.php?qn=1352) | 64.21 | 0.53 |
|  | MOL001353 | [GA60](http://tcmspw.com/molecule.php?qn=1353) | 93.17 | 0.53 |
|  | MOL001355 | [GA63](http://tcmspw.com/molecule.php?qn=1355) | 65.54 | 0.54 |
|  | MOL001358 | [gibberellin 7](http://tcmspw.com/molecule.php?qn=1358) | 73.8 | 0.5 |
|  | MOL001360 | [GA77](http://tcmspw.com/molecule.php?qn=1360) | 87.89 | 0.53 |
|  | MOL001361 | [GA87](http://tcmspw.com/molecule.php?qn=1361) | 68.85 | 0.57 |
|  | MOL001368 | [3-O-p-coumaroylquinic acid](http://tcmspw.com/molecule.php?qn=1368) | 37.63 | 0.29 |
|  | MOL001371 | [Populoside_qt](http://tcmspw.com/molecule.php?qn=1371) | 108.89 | 0.2 |
|  | MOL000296 | [hederagenin](http://tcmspw.com/molecule.php?qn=296) | 36.91 | 0.75 |
|  | MOL000358 | [beta-sitosterol](http://tcmspw.com/molecule.php?qn=358) | 36.91 | 0.75 |
|  | MOL000493 | [campesterol](http://tcmspw.com/molecule.php?qn=493) | 37.58 | 0.71 |
|  | MOL001320 | [Amygdalin](http://tcmspw.com/molecule.php?qn=1320) | 4.42 | 0.61 |

Table S5:The therapeutic target corresponding to each component in RP

| **Herb** | **Molecule name** | **Target name** |
| --- | --- | --- |
| Rhubarb | EUPATIN | Nitric oxide synthase, inducible |
|  | EUPATIN | Androgen receptor |
|  | EUPATIN | Coagulation factor Xa |
|  | EUPATIN | Prostaglandin G/H synthase 2 |
|  | EUPATIN | Coagulation factor VII |
|  | EUPATIN | DNA topoisomerase II |
|  | EUPATIN | Estrogen receptor beta |
|  | EUPATIN | Dipeptidyl peptidase IV |
|  | EUPATIN | Heat shock protein HSP 90 |
|  | EUPATIN | Trypsin-1 |
|  | EUPATIN | Nuclear receptor coactivator 2 |
|  | EUPATIN | Calmodulin |
|  | EUPATIN | Thrombin |
|  | EUPATIN | Sodium channel protein type 5 subunit alpha |
|  | EUPATIN | Vascular endothelial growth factor receptor 2 |
|  | EUPATIN | Peroxisome proliferator activated receptor delta |
|  | Mutatochrome |  |
|  | Physciondiglucoside | DNA topoisomerase II |
|  | Procyanidin B-5,3'-O-gallate |  |
|  | rhein | Prostaglandin G/H synthase 1 |
|  | rhein | Prostaglandin G/H synthase 2 |
|  | rhein | Heat shock protein HSP 90 |
|  | rhein | Phosphatidylinositol-4,5-bisphosphate 3-kinase catalytic subunit, gamma isoform |
|  | rhein | Nuclear receptor coactivator 2 |
|  | rhein | Aldose reductase |
|  | rhein | Transcription factor AP-1 |
|  | Sennoside E_qt |  |
|  | Torachrysone-8-O-beta-D-(6'-oxayl)-glucoside | DNA topoisomerase II |
|  | Toralactone | Nitric oxide synthase, inducible |
|  | Toralactone | Prostaglandin G/H synthase 1 |
|  | Toralactone | Estrogen receptor |
|  | Toralactone | Prostaglandin G/H synthase 2 |
|  | Toralactone | Estrogen receptor beta |
|  | Toralactone | Heat shock protein HSP 90 |
|  | Toralactone | Phosphatidylinositol-4,5-bisphosphate 3-kinase catalytic subunit, gamma isoform |
|  | Toralactone | Serine/threonine-protein kinase Chk1 |
|  | Toralactone | mRNA of PKA Catalytic Subunit C-alpha |
|  | Emodin-1-O-beta-D-glucopyranoside | DNA topoisomerase II |
|  | Sennoside D_qt |  |
|  | Daucosterol_qt | Progesterone receptor |
|  | Daucosterol_qt | Nuclear receptor coactivator 2 |
|  | palmidin A |  |
|  | beta-sitosterol | Progesterone receptor |
|  | beta-sitosterol | Nuclear receptor coactivator 2 |
|  | beta-sitosterol | Prostaglandin G/H synthase 1 |
|  | beta-sitosterol | Prostaglandin G/H synthase 2 |
|  | beta-sitosterol | Heat shock protein HSP 90 |
|  | beta-sitosterol | Phosphatidylinositol-4,5-bisphosphate 3-kinase catalytic subunit, gamma isoform |
|  | beta-sitosterol | Potassium voltage-gated channel subfamily H member 2 |
|  | beta-sitosterol | mRNA of PKA Catalytic Subunit C-alpha |
|  | beta-sitosterol | Dopamine D1 receptor |
|  | beta-sitosterol | Muscarinic acetylcholine receptor M3 |
|  | beta-sitosterol | Muscarinic acetylcholine receptor M1 |
|  | beta-sitosterol | Sodium channel protein type 5 subunit alpha |
|  | beta-sitosterol | Gamma-aminobutyric-acid receptor alpha-2 subunit |
|  | beta-sitosterol | Muscarinic acetylcholine receptor M4 |
|  | beta-sitosterol | CGMP-inhibited 3',5'-cyclic phosphodiesterase A |
|  | beta-sitosterol | 5-hydroxytryptamine 2A receptor |
|  | beta-sitosterol | Gamma-aminobutyric-acid receptor alpha-5 subunit |
|  | beta-sitosterol | Alpha-1A adrenergic receptor |
|  | beta-sitosterol | Gamma-aminobutyric-acid receptor alpha-3 subunit |
|  | beta-sitosterol | Muscarinic acetylcholine receptor M2 |
|  | beta-sitosterol | Alpha-1B adrenergic receptor |
|  | beta-sitosterol | Beta-2 adrenergic receptor |
|  | beta-sitosterol | Neuronal acetylcholine receptor subunit alpha-2 |
|  | beta-sitosterol | Sodium-dependent serotonin transporter |
|  | beta-sitosterol | Mu-type opioid receptor |
|  | beta-sitosterol | Gamma-aminobutyric acid receptor subunit alpha-1 |
|  | beta-sitosterol | Neuronal acetylcholine receptor protein, alpha-7 chain |
|  | beta-sitosterol | Cytochrome P450-cam |
|  | beta-sitosterol | Apoptosis regulator Bcl-2 |
|  | beta-sitosterol | Apoptosis regulator BAX |
|  | beta-sitosterol | Caspase-9 |
|  | beta-sitosterol | Transcription factor AP-1 |
|  | beta-sitosterol | Caspase-3 |
|  | beta-sitosterol | Caspase-8 |
|  | beta-sitosterol | Protein kinase C alpha type |
|  | beta-sitosterol | Transforming growth factor beta-1 |
|  | beta-sitosterol | Serum paraoxonase/arylesterase 1 |
|  | beta-sitosterol | Microtubule-associated protein 2 |
|  | aloe-emodin | Prostaglandin G/H synthase 1 |
|  | aloe-emodin | Prostaglandin G/H synthase 2 |
|  | aloe-emodin | Heat shock protein HSP 90 |
|  | aloe-emodin | Phosphatidylinositol-4,5-bisphosphate 3-kinase catalytic subunit, gamma isoform |
|  | aloe-emodin | mRNA of PKA Catalytic Subunit C-alpha |
|  | aloe-emodin | Nuclear receptor coactivator 2 |
|  | aloe-emodin | cAMP-dependent protein kinase inhibitor alpha |
|  | aloe-emodin | Aldose reductase |
|  | aloe-emodin | Ig gamma-1 chain C region |
|  | aloe-emodin | Cyclin-dependent kinase inhibitor 1 |
|  | aloe-emodin | Eukaryotic translation initiation factor 6 |
|  | aloe-emodin | Apoptosis regulator BAX |
|  | aloe-emodin | Tumor necrosis factor |
|  | aloe-emodin | Caspase-3 |
|  | aloe-emodin | Cellular tumor antigen p53 |
|  | aloe-emodin | Fatty acid synthase |
|  | aloe-emodin | Protein kinase C alpha type |
|  | aloe-emodin | Protein kinase C epsilon type |
|  | aloe-emodin | Cell division control protein 2 homolog |
|  | aloe-emodin | Proliferating cell nuclear antigen |
|  | aloe-emodin | Myc proto-oncogene protein |
|  | aloe-emodin | Interleukin-1 beta |
|  | aloe-emodin | Protein kinase C delta type |
|  | aloe-emodin | G2/mitotic-specific cyclin-B1 |
|  | gallic acid-3-O-(6'-O-galloyl)-glucoside |  |
|  | (-)-catechin | Prostaglandin G/H synthase 1 |
|  | (-)-catechin | Estrogen receptor |
|  | (-)-catechin | Prostaglandin G/H synthase 2 |
|  | (-)-catechin | Heat shock protein HSP 90 |
|  | (-)-catechin | Beta-lactamase |
|  | (-)-catechin | mRNA of PKA Catalytic Subunit C-alpha |
|  | (-)-catechin | Nuclear receptor coactivator 2 |
|  | (-)-catechin | Calmodulin |
|  | (-)-catechin | Fatty acid synthase |
|  | (-)-catechin | Peroxisome proliferator-activated receptor gamma |
|  | (-)-catechin | Krueppel-like factor 7 |
|  | emodin | Prostaglandin G/H synthase 1 |
|  | emodin | Prostaglandin G/H synthase 2 |
|  | emodin | Coagulation factor VII |
|  | emodin | Heat shock protein HSP 90 |
|  | emodin | Phosphatidylinositol-4,5-bisphosphate 3-kinase catalytic subunit, gamma isoform |
|  | emodin | mRNA of PKA Catalytic Subunit C-alpha |
|  | emodin | Ig gamma-1 chain C region |
|  | emodin | Coagulation factor Xa |
|  | emodin | Vascular endothelial growth factor receptor 2 |
|  | emodin | DNA topoisomerase II |
|  | emodin | Nuclear receptor coactivator 2 |
|  | emodin | Nuclear receptor coactivator 1 |
|  | emodin | Calmodulin |
|  | emodin | Cyclin-dependent kinase inhibitor 1 |
|  | emodin | Vascular endothelial growth factor receptor 1 |
|  | emodin | Matrix metalloproteinase-9 |
|  | emodin | Pro-epidermal growth factor |
|  | emodin | Tumor necrosis factor |
|  | emodin | Caspase-3 |
|  | emodin | Cellular tumor antigen p53 |
|  | emodin | Protein kinase C epsilon type |
|  | emodin | Interstitial collagenase |
|  | emodin | Peroxisome proliferator-activated receptor gamma |
|  | emodin | Myc proto-oncogene protein |
|  | emodin | Cytochrome P450 1A1 |
|  | emodin | Interleukin-1 beta |
|  | emodin | Protein kinase C delta type |
|  | emodin | Granulocyte-macrophage colony-stimulating factor |
|  | emodin | Transforming growth factor beta-1 |
|  | emodin | Actin, aortic smooth muscle |
|  | emodin | Amine oxidase [flavin-containing] B |
|  | emodin | Tyrosine-protein kinase BTK |
|  | emodin | Solute carrier family 2, facilitated glucose transporter member 4 |
|  | emodin | Vascular endothelial growth factor receptor 3 |
|  | emodin | Solute carrier family 2, facilitated glucose transporter member 1 |
|  | Crysophanol | Prostaglandin G/H synthase 1 |
|  | Crysophanol | Prostaglandin G/H synthase 2 |
|  | Crysophanol | Heat shock protein HSP 90 |
|  | Crysophanol | Nuclear receptor coactivator 2 |
|  | Crysophanol | Calmodulin |
|  | Crysophanol | Sodium channel protein type 5 subunit alpha |
|  | Crysophanol | CGMP-inhibited 3',5'-cyclic phosphodiesterase A |
|  | Crysophanol | Gamma-aminobutyric acid receptor subunit alpha-1 |
|  | Crysophanol | Phosphatidylinositol-4,5-bisphosphate 3-kinase catalytic subunit, gamma isoform |
|  | Crysophanol | mRNA of PKA Catalytic Subunit C-alpha |
|  | Crysophanol | Ig gamma-1 chain C region |
|  | Crysophanol | cAMP-dependent protein kinase inhibitor alpha |
|  | Physcion | Prostaglandin G/H synthase 1 |
|  | Physcion | Sodium channel protein type 5 subunit alpha |
|  | Physcion | Prostaglandin G/H synthase 2 |
|  | Physcion | Nitric-oxide synthase, endothelial |
|  | Physcion | Coagulation factor VII |
|  | Physcion | DNA topoisomerase II |
|  | Physcion | Heat shock protein HSP 90 |
|  | Physcion | Phosphatidylinositol-4,5-bisphosphate 3-kinase catalytic subunit, gamma isoform |
|  | Physcion | Beta-lactamase |
|  | Physcion | Ig gamma-1 chain C region |
|  | Physcion | Nuclear receptor coactivator 2 |
|  | Physcion | Nuclear receptor coactivator 1 |
|  | Physcion | cAMP-dependent protein kinase inhibitor alpha |
|  | Physcion | Calmodulin |
|  | Physcion | mRNA of PKA Catalytic Subunit C-alpha |
|  | Physcion | Coagulation factor Xa |
|  | Physcion | Retinoic acid receptor RXR-alpha |
| Peach kernel | Sitosterol alpha1 | Progesterone receptor |
|  | Sitosterol alpha1 | Prostaglandin G/H synthase 2 |
|  | Sitosterol alpha1 | Gamma-aminobutyric acid receptor subunit alpha-1 |
|  | Sitosterol alpha1 | Alcohol dehydrogenase 1C |
|  | Sitosterol alpha1 | Cytochrome P450-cam |
|  | Sitosterol alpha1 | Mineralocorticoid receptor |
|  | 2,3-didehydro GA70 | Prostaglandin G/H synthase 1 |
|  | 2,3-didehydro GA70 | Muscarinic acetylcholine receptor M1 |
|  | 2,3-didehydro GA70 | Prostaglandin G/H synthase 2 |
|  | 2,3-didehydro GA70 | Sodium-dependent noradrenaline transporter |
|  | 2,3-didehydro GA70 | Gamma-aminobutyric acid receptor subunit alpha-1 |
|  | 2,3-didehydro GA70 | Trypsin-1 |
|  | 2,3-didehydro GA70 | Glutamate receptor 2 |
|  | 2,3-didehydro GA77 | Prostaglandin G/H synthase 2 |
|  | 2,3-didehydro GA77 | Carbonic anhydrase II |
|  | 2,3-didehydro GA77 | Gamma-aminobutyric acid receptor subunit alpha-1 |
|  | 2,3-didehydro GA77 | Nuclear receptor coactivator 2 |
|  | 2,3-didehydro GA77 | Gamma-aminobutyric-acid receptor subunit alpha-6 |
|  | GA119 | Cytochrome P450-cam |
|  | GA120 | Muscarinic acetylcholine receptor M3 |
|  | GA120 | Muscarinic acetylcholine receptor M1 |
|  | GA120 | Prostaglandin G/H synthase 2 |
|  | GA120 | Gamma-aminobutyric-acid receptor alpha-3 subunit |
|  | GA120 | Muscarinic acetylcholine receptor M2 |
|  | GA120 | Gamma-aminobutyric acid receptor subunit alpha-1 |
|  | GA120 | Neuronal acetylcholine receptor protein, alpha-7 chain |
|  | GA120 | Gamma-aminobutyric-acid receptor subunit alpha-6 |
|  | GA121-isolactone | Progesterone receptor |
|  | GA122 |  |
|  | GA122-isolactone | Progesterone receptor |
|  | gibberellin 17 |  |
|  | 4a-formyl-7alpha-hydroxy-1-methyl-8-methylidene-4aalpha,4bbeta-gibbane-1alpha,10beta-dicarboxylic acid | Mineralocorticoid receptor |
|  | 4a-formyl-7alpha-hydroxy-1-methyl-8-methylidene-4aalpha,4bbeta-gibbane-1alpha,10beta-dicarboxylic acid | Progesterone receptor |
|  | GA30 |  |
|  | Gibberellin A44 | Mineralocorticoid receptor |
|  | Gibberellin A44 | Gamma-aminobutyric acid receptor subunit alpha-1 |
|  | Gibberellin A44 | Gamma-aminobutyric-acid receptor subunit alpha-6 |
|  | GA54 | Coagulation factor Xa |
|  | GA54 | Prostaglandin G/H synthase 2 |
|  | GA54 | mRNA of Protein-tyrosine phosphatase, non-receptor type 1 |
|  | GA54 | Heat shock protein HSP 90 |
|  | GA54 | Nuclear receptor coactivator 2 |
|  | GA54 | Calmodulin |
|  | GA60 | Gamma-aminobutyric-acid receptor alpha-2 subunit |
|  | GA60 | Gamma-aminobutyric-acid receptor alpha-3 subunit |
|  | GA60 | Muscarinic acetylcholine receptor M2 |
|  | GA60 | Gamma-aminobutyric acid receptor subunit alpha-1 |
|  | GA60 | Glutamate receptor 2 |
|  | GA63 | Prostaglandin G/H synthase 2 |
|  | GA63 | Gamma-aminobutyric acid receptor subunit alpha-1 |
|  | GA63 | Neuronal acetylcholine receptor protein, alpha-7 chain |
|  | GA63 | Glutamate receptor 2 |
|  | GA63 | Gamma-aminobutyric-acid receptor subunit alpha-6 |
|  | gibberellin 7 | Muscarinic acetylcholine receptor M3 |
|  | gibberellin 7 | Muscarinic acetylcholine receptor M1 |
|  | gibberellin 7 | Prostaglandin G/H synthase 2 |
|  | gibberellin 7 | CGMP-inhibited 3',5'-cyclic phosphodiesterase A |
|  | gibberellin 7 | Sodium-dependent dopamine transporter |
|  | gibberellin 7 | Beta-2 adrenergic receptor |
|  | gibberellin 7 | Sodium-dependent serotonin transporter |
|  | GA77 | Gamma-aminobutyric-acid receptor alpha-2 subunit |
|  | GA77 | Gamma-aminobutyric acid receptor subunit alpha-1 |
|  | GA77 | Cytochrome P450-cam |
|  | GA77 | Glutamate receptor 2 |
|  | GA87 | Prostaglandin G/H synthase 2 |
|  | GA87 | Carbonic anhydrase II |
|  | 3-O-p-coumaroylquinic acid | Prostaglandin G/H synthase 1 |
|  | 3-O-p-coumaroylquinic acid | Prostaglandin G/H synthase 2 |
|  | 3-O-p-coumaroylquinic acid | mRNA of Protein-tyrosine phosphatase, non-receptor type 1 |
|  | 3-O-p-coumaroylquinic acid | Heat shock protein HSP 90 |
|  | 3-O-p-coumaroylquinic acid | Phosphatidylinositol-4,5-bisphosphate 3-kinase catalytic subunit, gamma isoform |
|  | 3-O-p-coumaroylquinic acid | mRNA of PKA Catalytic Subunit C-alpha |
|  | 3-O-p-coumaroylquinic acid | Nuclear receptor coactivator 2 |
|  | 3-O-p-coumaroylquinic acid | Calmodulin |
|  | Populoside_qt |  |
|  | hederagenin | Progesterone receptor |
|  | hederagenin | Nuclear receptor coactivator 2 |
|  | hederagenin | Muscarinic acetylcholine receptor M3 |
|  | hederagenin | Muscarinic acetylcholine receptor M1 |
|  | hederagenin | Gamma-aminobutyric-acid receptor alpha-2 subunit |
|  | hederagenin | Gamma-aminobutyric-acid receptor alpha-3 subunit |
|  | hederagenin | Muscarinic acetylcholine receptor M2 |
|  | hederagenin | Alpha-1B adrenergic receptor |
|  | hederagenin | Gamma-aminobutyric acid receptor subunit alpha-1 |
|  | hederagenin | Glutamate receptor 2 |
|  | hederagenin | Gamma-aminobutyric-acid receptor subunit alpha-6 |
|  | hederagenin | Gamma-aminobutyric-acid receptor alpha-5 subunit |
|  | hederagenin | Ig gamma-1 chain C region |
|  | hederagenin | Alcohol dehydrogenase 1B |
|  | hederagenin | Alcohol dehydrogenase 1C |
|  | hederagenin | Lysozyme |
|  | hederagenin | Nicotinate-nucleotide--dimethylbenzimidazole phosphoribosyltransferase |
|  | hederagenin | Prostaglandin G/H synthase 1 |
|  | hederagenin | Sodium channel protein type 5 subunit alpha |
|  | hederagenin | Prostaglandin G/H synthase 2 |
|  | hederagenin | Retinoic acid receptor RXR-alpha |
|  | hederagenin | CGMP-inhibited 3',5'-cyclic phosphodiesterase A |
|  | hederagenin | Sodium-dependent noradrenaline transporter |
|  | hederagenin | Cytochrome P450-cam |
|  | beta-sitosterol | Progesterone receptor |
|  | beta-sitosterol | Nuclear receptor coactivator 2 |
|  | beta-sitosterol | Prostaglandin G/H synthase 1 |
|  | beta-sitosterol | Prostaglandin G/H synthase 2 |
|  | beta-sitosterol | Heat shock protein HSP 90 |
|  | beta-sitosterol | Phosphatidylinositol-4,5-bisphosphate 3-kinase catalytic subunit, gamma isoform |
|  | beta-sitosterol | Potassium voltage-gated channel subfamily H member 2 |
|  | beta-sitosterol | mRNA of PKA Catalytic Subunit C-alpha |
|  | beta-sitosterol | Dopamine D1 receptor |
|  | beta-sitosterol | Muscarinic acetylcholine receptor M3 |
|  | beta-sitosterol | Muscarinic acetylcholine receptor M1 |
|  | beta-sitosterol | Sodium channel protein type 5 subunit alpha |
|  | beta-sitosterol | Gamma-aminobutyric-acid receptor alpha-2 subunit |
|  | beta-sitosterol | Muscarinic acetylcholine receptor M4 |
|  | beta-sitosterol | CGMP-inhibited 3',5'-cyclic phosphodiesterase A |
|  | beta-sitosterol | 5-hydroxytryptamine 2A receptor |
|  | beta-sitosterol | Gamma-aminobutyric-acid receptor alpha-5 subunit |
|  | beta-sitosterol | Alpha-1A adrenergic receptor |
|  | beta-sitosterol | Gamma-aminobutyric-acid receptor alpha-3 subunit |
|  | beta-sitosterol | Muscarinic acetylcholine receptor M2 |
|  | beta-sitosterol | Alpha-1B adrenergic receptor |
|  | beta-sitosterol | Beta-2 adrenergic receptor |
|  | beta-sitosterol | Neuronal acetylcholine receptor subunit alpha-2 |
|  | beta-sitosterol | Sodium-dependent serotonin transporter |
|  | beta-sitosterol | Mu-type opioid receptor |
|  | beta-sitosterol | Gamma-aminobutyric acid receptor subunit alpha-1 |
|  | beta-sitosterol | Neuronal acetylcholine receptor protein, alpha-7 chain |
|  | beta-sitosterol | Cytochrome P450-cam |
|  | beta-sitosterol | Apoptosis regulator Bcl-2 |
|  | beta-sitosterol | Apoptosis regulator BAX |
|  | beta-sitosterol | Caspase-9 |
|  | beta-sitosterol | Transcription factor AP-1 |
|  | beta-sitosterol | Caspase-3 |
|  | beta-sitosterol | Caspase-8 |
|  | beta-sitosterol | Protein kinase C alpha type |
|  | beta-sitosterol | Transforming growth factor beta-1 |
|  | beta-sitosterol | Serum paraoxonase/arylesterase 1 |
|  | beta-sitosterol | Microtubule-associated protein 2 |
|  | campesterol | Progesterone receptor |
|  | campesterol | Prostaglandin G/H synthase 1 |
|  | campesterol | Prostaglandin G/H synthase 2 |
|  | campesterol | Heat shock protein HSP 90 |
|  | campesterol | Phosphatidylinositol-4,5-bisphosphate 3-kinase catalytic subunit, gamma isoform |
|  | campesterol | Nuclear receptor coactivator 2 |
|  | Amygdalin | Prostaglandin G/H synthase 1 |
|  | Amygdalin | Prostaglandin G/H synthase 2 |
|  | Amygdalin | Heat shock protein HSP 90 |
|  | Amygdalin | Phosphatidylinositol-4,5-bisphosphate 3-kinase catalytic subunit, gamma isoform |
|  | Amygdalin | mRNA of PKA Catalytic Subunit C-alpha |
|  | Amygdalin | Nuclear receptor coactivator 2 |
|  | Amygdalin | Calmodulin |

Table S6:Common targets and Gene symbol of rhubarb and peach kernel

| **Target name** | **Gene symbol** |
| --- | --- |
| Nitric oxide synthase, inducible | NOS2 |
| Androgen receptor | AR |
| Coagulation factor Xa | F10 |
| Prostaglandin G/H synthase 2 | PTGS2 |
| Coagulation factor VII | F7 |
| DNA topoisomerase II | TOP2 |
| Estrogen receptor beta | ESR2 |
| Dipeptidyl peptidase IV | DPP4 |
| Heat shock protein HSP 90 | HSP90 |
| Trypsin-1 | PRSS1 |
| Nuclear receptor coactivator 2 | NCOA2 |
| Calmodulin | CAM |
| Thrombin | F2 |
| Sodium channel protein type 5 subunit alpha | SCN5A |
| Vascular endothelial growth factor receptor 2 | KDR |
| Peroxisome proliferator activated receptor delta | PPARD |
| Prostaglandin G/H synthase 1 | PTGS1 |
| Phosphatidylinositol-4,5-bisphosphate 3-kinase catalytic subunit, gamma isoform | PIK3CG |
| Aldose reductase | AKR1B1 |
| Transcription factor AP-1 | JUN |
| Estrogen receptor | ESR1 |
| Serine/threonine-protein kinase Chk1 | CHEK1 |
| mRNA of PKA Catalytic Subunit C-alpha | PKAC1 |
| Progesterone receptor | PGR |
| Potassium voltage-gated channel subfamily H member 2 | KCNH2 |
| Dopamine D1 receptor | DAD1 |
| Muscarinic acetylcholine receptor M3 | CHRM3 |
| Muscarinic acetylcholine receptor M1 | CHRM1 |
| Gamma-aminobutyric-acid receptor alpha-2 subunit | GABRA2 |
| Muscarinic acetylcholine receptor M4 | CHRM4 |
| CGMP-inhibited 3',5'-cyclic phosphodiesterase A | PDE3A |
| 5-hydroxytryptamine 2A receptor | HTR2A |
| Gamma-aminobutyric-acid receptor alpha-5 subunit | GABRA5 |
| Alpha-1A adrenergic receptor | ADRA1A |
| Gamma-aminobutyric-acid receptor alpha-3 subunit | GABRA3 |
| Muscarinic acetylcholine receptor M2 | CHRM2 |
| Alpha-1B adrenergic receptor | ADRA1B |
| Beta-2 adrenergic receptor | ADRB2 |
| Neuronal acetylcholine receptor subunit alpha-2 | CHRNA2 |
| Sodium-dependent serotonin transporter | ALC6A4 |
| Mu-type opioid receptor | OPRM1 |
| Gamma-aminobutyric acid receptor subunit alpha-1 | GABRA1 |
| Neuronal acetylcholine receptor protein, alpha-7 chain | CHRNA7 |
| Cytochrome P450-cam | CYP101A1 |
| Apoptosis regulator Bcl-2 | BCL2 |
| Apoptosis regulator BAX | BAX |
| Caspase-9 | CASP9 |
| Caspase-3 | CASP3 |
| Caspase-8 | CASP8 |
| Protein kinase C alpha type | PRKCA |
| Transforming growth factor beta-1 | TGFB1 |
| Serum paraoxonase/arylesterase 1 | PON1 |
| Microtubule-associated protein 2 | MAP2 |
| cAMP-dependent protein kinase inhibitor alpha | PKIA |
| Ig gamma-1 chain C region | IGHG1 |
| Cyclin-dependent kinase inhibitor 1 | CDKN1A |
| Eukaryotic translation initiation factor 6 | EIF6 |
| Tumor necrosis factor | TNF |
| Cellular tumor antigen p53 | TP53 |
| Fatty acid synthase | FASN |
| Protein kinase C epsilon type | PRKCE |
| Cell division control protein 2 homolog | CRK2 |
| Proliferating cell nuclear antigen | PCNA |
| Myc proto-oncogene protein | MYC |
| Interleukin-1 beta | IL1B |
| Protein kinase C delta type | PRKCD |
| G2/mitotic-specific cyclin-B1 | CCNB1 |
| Beta-lactamase | AMPC |
| Peroxisome proliferator-activated receptor gamma | PPARG |
| Krueppel-like factor 7 | KLF7 |
| Nuclear receptor coactivator 1 | NCOA1 |
| Vascular endothelial growth factor receptor 1 | VEGFR1 |
| Matrix metalloproteinase-9 | MMP9 |
| Pro-epidermal growth factor | EGF |
| Interstitial collagenase | MMP1 |
| Cytochrome P450 1A1 | CYP1A1 |
| Granulocyte-macrophage colony-stimulating factor | GM-CSF |
| Actin, aortic smooth muscle | ACTA2 |
| Amine oxidase [flavin-containing] B | MAOB |
| Tyrosine-protein kinase BTK | BTK |
| Solute carrier family 2, facilitated glucose transporter member 4 | SLC2A4 |
| Vascular endothelial growth factor receptor 3 | VEGFR3 |
| Solute carrier family 2, facilitated glucose transporter member 1 | SLC2A1 |
| Alcohol dehydrogenase 1C | ADH1C |
| Mineralocorticoid receptor | NR3C2 |
| Sodium-dependent noradrenaline transporter | SLC6A2 |
| Glutamate receptor 2 | GRIA2 |
| Carbonic anhydrase II | CA2 |
| Gamma-aminobutyric-acid receptor subunit alpha-6 | GABRA6 |
| mRNA of Protein-tyrosine phosphatase, non-receptor type 1 | PTPN1 |
| Sodium-dependent dopamine transporter | SLC6A3 |
| Alcohol dehydrogenase 1B | ADH1B |
| Lysozyme | LYZ |
| Nicotinate-nucleotide--dimethylbenzimidazole phosphoribosyltransferase | COBT |
| Retinoic acid receptor RXR-alpha | RXRA |
| Nitric-oxide synthase, endothelial | NOS3 |

Table S7 Gene symbol corresponding to adenomyosis

| **GenaCard database** | **OMIM database** |
| --- | --- |
| ENDO1， CYP19A1， PGR， VEGFA， ESR1， GNRH1， IL6， ESR2， PTGS2， HOXA10， CXCL8， MMP2， GSTM1， CCL2， IL1B， PTEN， MUC16， CDKN2B-AS1， MMP3， CYP1A1， NR5A1， CYP17A1， WNT4， KDR， MMP1， CCL5， AHRR， IL1R2， LIF， HGF， PAEP， HSD17B1， AHR， RXFP1， PLAU， AHSG， CYP1B1， MMP7， MIF， EMX2， HNF1B， GNRHR， STX5， CXCL5， STS， STAR， TIMP1， ENO1， IGFBP1， MIR145， MIR29C， NR2F2， MIR126， MIR20A， CCN1， CALB2， MIR199A1， LEFTY2， H19， MIR196B， MIR200B， MIR141， MIR143， MIR34C， MIR99A， MIR200A， MIR125A， MIR142， MIR150， MIR100， MIR223， MIR99B， MIR424， RN7SL7P， TP53， MMP9， CDH1， MME， CTNNB1， TGFB1， ARID1A， INHBA， PRL， TIMP2， NGF， SPP1， TNF， ERBB2， AMH， KRAS， OXTR， CD44， THOC6， PAK4， IL10， BAX， KRT7， EGF， AKT1， EGFR， COMT， CDKN2A， MUC1， PIK3CA， PAX2， CCND1， OXT， GSTT1， ICAM1， MIR17， CPT2， USH2A， MLH1， KRT20， BRCA1， BRCA2， SMAD4， WT1， IFNG， LEP， MYC， IL1A， VIM， TOP2A， MCM2， MCM7， OPRM1， MIR9-1， MIR34B， MIR30E， MIR361， IL2， MIR22， PPP2R1A， MIR196A1， MIR214， MIR542， BCL2， MIR34A， IL1R1， IL33， MSLN， MIRLET7A1， MIR200C， LOC108491836， HRAS， BDKRB1， MIR23B， TLR4， CDKN1B， CNR1， GPER1， TNFRSF1B， HIF1A， APC， MIR221， CXCL12， FAS， HLA-DRB1， PTPN22， PTPRF， SERPINA3， H2AC18， MIR30A， MIR629， TTTY5， TTTY8， TTTY22， CHL1-AS2， TTTY23， TTTY21， TTTY4C， CHL1-AS1， IL15， VEZT， IL4， NAT2， CCR1， HOXA11， AR， FASLG， FSHR， MIR331， MIR574， IL18， GC， PPARG， LOC110386951， CDKN1A， FOXP3， CSF1， IL37， HP， GNRHR2， GREB1， CXCL1， PAX8， PCNA， ACE， FCRL3， IL1RN， LHB， CRH， CYP2C19， ARNT， IGFBP3， XRCC1， NTRK2， BSG， GSTP1， IGF1， UCN， TNFRSF11B， BMP6， BIRC5， LTA， CGA， IL16， SERPINE1， MAPK1， IGF1R， MDM2， CCNE1， NOS3， RELA， LEPR， NFKB1， IGF2， HLA-G， TNFRSF1A， CDX2， MIR21， FGF2， FN1， IL17A， TERT， BDNF， HLA-A， IL11， HSD17B2， HLA-B， TRPV1， GALT， HPSE， ACP1， BRAF， MET， FST， CCL11， FLT1， STAT3， NPY， ACTC1， ATM， GNAS， SMARCA4， CASP3， PPM1D， GAPDH， CACNA1B， MSH2， CCR6， MAPK8IP1， TPSAB1， CEACAM5， CDK12， U2AF1， DLG2， ACSBG1， LCOR， VGF， C1RL， RNF43， MAPK8IP2， PRDM10， SERPINB11， ESS2， ATPSCKMT， MIR146A， MIR27A， MIR30C1， MIR106B， MIR10A， MIR132， MIR29A， MIR140， MIR149， MIR93， MIR373， MIRLET7E， MIR181C， MIR127， MIR193B， MIR92A1， MIR483， MIR491， MIR181A1， MIR101-1， MIR182， MIR205， MIR139， MIR25， MIR31， MIR520A， MIR590， MIR423， MIR103A1， MIR1307， MIR15B， MIR186， MIR18A， MIR19A， MIR218-1， MIR193A， MIR675， MIR338， MIR518F， MIR512-1， MIR486-1， MIR371A， MIR224， MIR526B， MIR144， MIR744， MIR517A， MIR15A， MIR20B， OVAAL， VCAM1， PTGER4， CSF2， GHRL， CXCR4， MIRLET7B， MTOR， PROK1， MKI67， C3， MAPK3， OGG1， KLF9， IL13， FKBP4， IL1RAP， BGLAP， VEGFC， NOTCH1， DES， BCL2L1， ITGB3， SERPINF1， CXCL10， CLDN4， ARSH， NTRK1， IL1RAPL2， DRD2， CD40LG， CFL1， BCL6， EZR， TAGLN， SOD1， SMAD2， TNFSF13B， PDE1C， FSHB， ITPK1， RND3， KSR2， ID4， ZBTB40， SORCS1， MOAP1， CSMD1， RNF144B， RBM43， OR1D2， OR1G1， HNRNPA3P1， LOC100287015， LOC100506885， TNFSF10， IL5， VIP， ANGPT2， TNFRSF10B， SULT1E1， NODAL， MPO， CD82， CDH2， C5， LTF， DNMT3A， TH， SERPINC1， DEFA1， ADIPOQ， MUC4， FGFR2， VDR， IDO1， ITGA6， TWIST1， KISS1， NGFR， NFE2L3， DEFA10P， ADH5P2， S100A4， TIMP3， SRC， MMP14， DNMT1， AMHR2， COX5A， ADM， F2RL1， CXCR2， TDGF1， FGF1， CDC42， CDKN2B， PTPRD， CKAP2L， RHOU， LINC00339， AKR1C3， TYMP， JUN， F2， IL6R， PTK2， LGALS3， SEMA3C， AXL， MIR191， WFDC2， SHBG， CCL21， CCL3， AGTR1， HMGA2， KITLG， CCL19， STAT4， CAT， ADAM17， ANXA1， SMAD3， ELANE， PHB， APEX1， SYP， MIRLET7F1， HMOX1， CD40， CTLA4， IL1RL1， ANGPT1， CDH3， CALCA， ADA， CLU， CXCR3， HLA-DQA1， ID2， IGFBP4， STIP1， PF4， KLF11， THBS1， IFNA2， ENO2， PARK7， HLA-C， LGR5， PTGES， XRCC3， CLDN3， NR1H2， PRDM1， KRT19， IL12B， PAPPA， PDCD6， GJA1， TSLP， NECTIN4， RAF1， MMP13， HDAC1， PGM1， CCNB1， CASP9， CRP， CDK1， CD79A， SOX2， SLC18A3， ANG， HMGB1， TET2， TET3， TET1， SIRT1， IL2RB， MST1R， GDF9， UCHL1， NME1， AGER， HLA-DPB1， RETN， XRCC4， SERPINA6， HTRA1， FOS， DNMT3B， CX3CL1， CCR5， IL6ST， CD69， ABCG2， CTSD， GATA6， DNMT3L， YBX1， CCNA2， POU5F1， MIR33B， CXCR1， FGFR1， SHC1， SLC6A4， PDGFA， SPINK1， PTP4A3， KIR2DS5， EBAG9， TMOD3， MIR451A， PDYN， AIF1， NFKBIA， BAD， HSD3B2， GH1， AGR2， S100P， SOD2， MAPK14， LHCGR， KIR3DL1， CCL25， KIR2DL3， CEBPA， CD163， CREB1， ITGAV， TACR1， LAMC2， TFF3， MUC2， SERPINA1， TNFRSF21， LOXL4， CD200， NR3C1， NOS2， PAK1， TGFBR1， CYP24A1， S100A6， SRD5A1， AKR1C1， LOC108863620， L1CAM， MICB， KLRK1， MICA， ULBP2， CYP3A4， LIPC， ELAVL1， GHRH， TPM3， CALD1， EFNA1， PIBF1， NPTX2， STOML2， TEK， CPB2， COPS5， FJX1， XDH， ANXA2， NR4A1， SERPINB2， MIR183， ALPP， NCOA1， CSF1R， DAPK1， NUMB， TXN， IRS2， TNFRSF10A， TXNIP， CCDC80， IRF5， TRAF1， CD36， GDF15， AFM， LEPQTL1， JAK1， PRKCB， NFE2L2， AKR1B1， PON1， MAP2K7， OSM， GCLC， CAMP， NANOG， NTN4， MT-CO2， DIRAS3， CYRIB， MIRLET7D， MIR122， MIRLET7F2， LOC110408762， EPCAM， BIRC2， BAK1， CSF3， TYK2， TGFB2， APOE， EIF4E， STK11， TSC2， RPS6KB1， NF1， RHEB， YAP1， EIF4EBP1， TGFB3， AXIN1， FEN1， F3， CCR7， STMN1， CD1A， PIAS3， STC1， IL32， CXCL11， RBP1， HOXB4， CXCL9， CD83， CRISPLD2， DEFA3， KIR2DL1， HLA-DRB4， MIR125B1， MIR196A2， MIR154， CRK， MEIS1， EZH2， ITGB1， GSK3B， STK4， CDC25A， KCNQ2， NRP1， CTCF， PRDX6， PRDX5， PTGS1， CRYAB， PROM1， SLIT2， AVP， FHIT， GREM1， KISS1R， ANXA4， FOXA2， AKAP13， CHIT1， CORO1A， PTN， NRP2， ETS2， TGFB1I1， SEMA3F， RHOC， CHD5， TAGLN2， NLRC5， LGALS9， UGT2B28， NUP210， FOXL2， PGRMC2， CCDC22， MAS1， RNH1， EIF3E， PCDH17， MUC17， VEGFD， USP17L2， HOXA11-AS， LOC110386948， PRKG1， TF， KRT18， GATA3， KLK3， SRA1， TLR2， CHUK， CASP1， EPOR， AGT， PLA2G6， HPGD， ALOX5， LMNB1， SLC11A2， ACTA2， CETP， PRDX2， ERCC2， MAF， ENG， CCR2， TAF1， ERCC5， F5， RORC， CLOCK， CHI3L1， PGRMC1， PGF， ID1， MFGE8， MDK， CAPN5， MAP1LC3A， BGN， BLMH， TNFRSF6B， UGCG， SMOC2， HSD17B7， EPO， CREB3L1， RPS23， SMPD3， PRELP， IL27， USF2， SGMS1， SLPI， MRC1， ILKAP， TRERF1， ERVW-1， KIR2DS4， MALAT1， MIR210， MIR148A， MIR449B， UBA52P1， KRT8， DUSP2， HDAC2， CASP8， AURKA， LIMK1， PLK1， EDNRA， AURKB， ITGA5， PLG， HMOX2， ISG15， LAMB1， VCAN， TNC， GATA2， CD55， EDN1， MTHFR， ROCK2， RHOA， CSF3R， ITGA2， ANTXR2， FKBP5， NCAM1， MYH11， TFDP1， LAMA1， USP10， SNAI2， NR5A2， MST1， COL18A1， BDKRB2， FCGR3A， MMP24， HLA-DQB1， SELE， SDC4， SNAI1， MMP12， LAMC1， KNG1， RPS27A， HAMP， TBX4， CLCN3， SLIT3， CD63， RECK， P2RX3， CCR9， ACKR3， CXCL6， RARRES1， PEMT， RARRES2， BNC2， IL25， S100A13， FSTL3， RACK1， PROKR1， IPO13， MRPL28， NCR1， TTC39B， EPRS1， MIR23A， TRB， MIR503， CTSB， BMPR1B， ESRRB， ACVR1， MGMT， APOA1， FOXO1， ACTN1， ITGA4， HSPD1， PPARGC1A， FPR1， CFI， PTGER3， PLA2G2A， FPR2， HMGCR， CRHR1， CHRNA7， MYOD1， CTSG， ACVR2A， EPHA3， LTB4R， CD86， MB， C9， LOXL1， CNTF， IL24， LILRB1， CD80， SNCG， MAP2， MTA1， CXCL13， KLRD1， MBD2， LTB4R2， LILRB2， KLRC1， PECAM1， MSI1， CNKSR1， ZFP36， MYOG， UCN3， UCN2， KIR3DS1， LINC00261， CDK6， COL1A1， STAT6， MAPK8， DPP4， CYP11A1， PTGIS， EPHX1， HMGA1， IGFBP7， FOLR1， ENPP1， DCN， AFP， GLO1， ALOX15， NAT1， CYP2E1， MSTN， ABCC4， WNT2， CCR8， MGST1， LGALS1， USF1， RUNX3， HSPA1A， DCLK1， ESD， CHGA， SERPINB3， CALB1， HPGDS， PHB2， S100A12， CCL1， AZU1， HPX， AMY2A， PSG2， SOX15， GNRH2， MIR520H， MIR2861， LOC110806262， ITGB2， IHH， MBL2， MCL1， DIABLO， F2R， AKR1C2， IL12RB1， UBC， SFRP4， POU1F1， NCOR1， NCOR2， PCSK5， RASSF2， RERG， NUDT6， INTS9， LINCMD1， ILK， POSTN， JAK3， ZEB1， PDCD1， ITGA3， FGA， IL11RA， CD274， SIK2， IL17F， CHL1， RLN1， PTGER2， MMP11， MIMT1， NRIP1， ETS1， TYRO3， FOXP1， GAS6， MIR381， CADM1， NECTIN3， CXCR6， CXCL16， CCL17， ARL4C， CCDC144NL-AS1， MAP3K1， CACNA1A， GRIN2D， MARK3， HLA-DRA， TLR1， MTAP， CDC73， C2， ATIC， XRCC5， MAP3K4， GPNMB， TLR6， SLC20A1， SMARCAL1， FOXP2， LAMC3， ALCAM， COL12A1， SYNE1， TLR10， NUP133， ATP8B1， PSMD13， SYNJ2， SKAP1， CYTH2， HNRNPF， KLF3， SNX9， ARID3B， CYP2W1， RHOJ， IL36G， CDH20， SERAC1， MACROD2， RMND1， ZBTB2， TFAP2D， LMTK3， DMRTA1， NAALADL2， FGD6， ARHGAP42， COX19， FAM114A1， AGBL4， CAPN14， TMEM143， SYNGR4， ZFAND2A， OR9Q1， CCN4， C1orf35， C7orf50， BEND5， COX8C， CCDC170， ARMT1， ETAA1， KIAA1549L， C18orf21， CALHM3， RFLNA， C14orf132， TMEM78， MROH5， CDC14C， C5orf67， MIR339， LINC02860， TUNAR， LINC02881， GUCY2EP， OR1E3， LINC00841， COX10-AS1， LINC00583， LINC00861， SNAP25-AS1， PLEKHM1P1， MEIS1-AS2， LOC646588， DUSP5P1， LINC01435， LINC01239， ZNF664-RFLNA， RSL24D1P11， MIR4418， LINC01365， TSBP1-AS1， LINC02612， SLC16A6P1， RNU6-786P， ENSG00000237838， HSPA8P15， ENSG00000237356， RNU6-808P， BNIP3P1， RAP1BP2， ENSG00000259621， ENSG00000254632， ENSG00000254532， ENSG00000266076， ENSG00000270933， LINC02674， LINC02500， ENSG00000271958， PARP1P2， ENSG00000225680， ENSG00000226920， ENSG00000228412， ENSG00000229116， PES1P2， ENSG00000235495， RNA5SP326， RNA5SP158， RN7SL318P， ENSG00000224079， ENSG00000259093， ENSG00000257943， ENSG00000260763， ENSG00000267098， ENSG00000265982， ENSG00000271399， ENSG00000277653， LINC02063， LINC02815， ENSG00000230695， SDAD1P2， ENSG00000228650， ENSG00000236921， ENSG00000237753， SNORD156-001， ENSG00000237590， ENSG00000287937， lnc-SPRED2-19， RNU6ATAC13P， RN7SL216P， RF00017-4630， HE856223， HE856005， lnc-NFE2L3-8， piR-46847-234， ENSG00000249943， JA662170， lnc-MTAP-3， RF00001-292， ENSG00000258595， ENSG00000271358， lnc-ZNF664-2， ENSG00000228064， piR-61514-138， ENSG00000234686， piR-48965-098， RF00017-7476， HSALNG0018001， lnc-FN1-4， lnc-ESR1-3， piR-43106-108， piR-43325-084， lnc-IL1A-5， LOC100506236， lnc-HNRNPA2B1-13， HSALNG0068725， HSALNG0011272， ENSG00000286122， RF00017-5953， lnc-RHOU-5， lnc-SERAC1-7， piR-41195-046， lnc-CCDC170-2， ENSG00000287410， 5MWI_A-088， ENSG00000285873， lnc-ATIC-10， ENSG00000286241， RF00017-7475， LOC105376845， RF00017-3044， ENSG00000258932， piR-38537-017， piR-31470-180， lnc-ZNF664-3， LOC105373868， ENSG00000288172， lnc-CDKN2B-4， lnc-COX7A2-1， piR-51974， piR-59769-059， ENSG00000287060， lnc-RND3-6， piR-39858-541， ENSG00000285409， ENSG00000286725， piR-34946-004， lnc-THRB-5， piR-30876-020， ENSG00000278256， HSALNG0118218， RF00994-254， ENSG00000243635， lnc-CDC42-3， FJ601684-081， HSALNG0024730， lnc-FGD6-5， HSALNG0019587， ENSG00000225356， HSALNG0077598， HSALNG0080495， lnc-RGS9-9， RF00017-3942， lnc-RGS9-10， GAP43， GJB1， GJB2， SKP2， CKS1B， CYP1A2， DFFA， DFFB， ARX， CRHBP， SSTR1， CD74， IGF2BP3， SSTR5， KIR2DL4， SGPP2， NR0B1， PLAUR， RB1， EPAS1， LIFR， TP63， ADIPOR1， ADIPOR2， CYCS， WNT7A， SGK1， UGT1A1， VWF， KRT17， LPAR2， ANGPTL1， CAPN7， BOK， LPAR4， OLFM4， CCL22， PIK3CG， NCOA2， AJAP1， TLR3， PRLR， XIAP， CYP27B1， GHSR， BIRC3， TDO2， CCR4， ARRB2， TCL1A， FGF9， ARRB1， GHRHR， TLN1， IL22， ZFP42， CGB5， MIR195， MIR363， ADAM10， NTRK3， MAP3K7， NOS1， PLAT， TGM2， INS， MAPK9， KDM1A， YWHAQ， PPIA， BECN1， AVPR1A， LCN2， INHA， SELENBP1， SDC1， CRHR2， AHSP， MIR10B， LINC-ROR， LOC106728418， HDAC3， LDLR， RAD50， PRKAA1， IRS1， ACP5， TTK， NCOA3， GNB1， SNRPN， CBR1， MED12， ELN， PDCD4， FSCN1， AGTR2， GNB2， GNB4， TIA1， APOA4， EEF1D， BCOR， PRPF8， GPT， CCT2， RPL8， CD68， EIF6， C4BPA， E2F6， HOXD9， CORO1C， OVGP1， NAT10， NCOA6， NFYA， AGFG1， HNRNPH2， CHD6， STOM， SYNCRIP， FIP1L1， UBXN11， MMP27， YTHDF1， ALYREF， AP2A2， MED9， HOXC10， MPRIP， FBXO45， PRR12， GCN1， CENPS-CORT， TFPI2， PIK3R1， SPARC， ALB， TTR， RXRA， KRT14， KRT5， MSN， SERPING1， SLC2A4， ACTN4， TGIF1， APOH， TOR1A， KRT4， IL18R1， ABCB7， KRT10， TIMP4， CEACAM3， MEGF10， IL18BP， PELP1， IBSP， TOR2A， PNOC， SCGB2A2， HSD3BP4， LOC110386947， TPO， CCR3， PTGER1， TCF21， LOC111188156， JAK2， FLT4， CREBBP， HCK， KAT2B， CTSH， BMP2， SLC7A11， NTF3， FBLN1， SELL， PPP1R13L， CHST4， FBXO5， H3C1， TGFBR2， MAPK10， PTPRC， NT5E， TNNI3， HSD11B1， POLE， CYP2D6， REN， GATA4， PPP2R1B， CYP3A5， TGFA， PPARA， ANXA5， ALDH3A2， AQP2， PDE4A， THY1， CD34， ELK1， CEBPB， TG， SMAD7， IL7， SP3， PDPN， MCAM， MED1， MLANA， HLX， LSR， MUC5AC， DLX4， TRO， SPA17， BCAS3， ANOS1， LBX1， LHX9， FNDC5， KLRA1P， NGF-AS1， LOC110366354 | BMIQ8， BULN， PFFE1， ST12， TUBB8， ZMYND11， DIP2C， LARP4B， GTPBP4， IDI2， IDI2AS1， IDI1， WDR37， ADARB2， PFKP， PITRM1， KLF6， MS2， AKR1E2， AKR1C1， AKR1C2， AKR1C3， AKR1C4， UCN3， NET1， CALML5， CALML3， LASTR， ASB13， GDI2， ANKRD16， FBXO18， IL15RA， IL2RA， RBM17， PFKFB3， PRKCQ， ARVD6， CRCS5， DGCR2， SFMBT2， ITIH5， ITIH2， KIN， ATP5F1C， TAF3， GATA3， CELF2， USP6NL， UPF2， DHTKD1， SEC61A2， NUDT5， CDC123， AD7， LPRS， CAMK1D， OPTN， MCM10， PHYH， SEPHS1， PRPF18， FRMD4A， ARMETL1， HSPA14， SUV39H2， DCLRE1C， MEIG1， RPP38， NMT2， ITGA8， MINDY3， PTER， C1QL3， RSU1， CUBN， TRDMT1， VIMAS1， VIM， ST8SIA6， HACD1， STAM， MRC1， SLC39A12， CACNB2， NSUN6， ARL5B， MALRD1， PLXDC2， NEBL， MIR1915， AF10， DNAJC1， EBLN1， COMMD3， BMI1， SPAG6， PIP4K2A， ARMC3， MSRB2， PTF1A， OTUD1， KIAA1217， ARHGAP21， PRTFDC1， THNSL1， ENKUR， GPR158， MYO3A， GAD2， APBB1IP， PDSS1， ABI1， ANKRD26， YME1L1， MASTL， ACBD5， PTCHD3， RAB18， MKX， ODAD2， MPP7， WAC， BAMBI， DFNB33， SVIL， KIAA1462， MTPAP， MAP3K8， LYZL2， EIG5， ZEB1AS1， ZEB1， ARHGAP12， KIF5B， EPC1， CCDC7， ITGB1， NRP1， PARD3， USH1K， CUL2， CREM， CCNY， GJD4， FZD8， ANKRD30A， ZNF25， ZNF33A， ZNF37A， AITD4， STHAG5， ZNF33B， BMS1， RET， CSGALNACT2， RASFEF1A， FXYD4， HNRPF， ZNF239， ZNF32， CXCL12， RASSF4， DEPP1， ZNF22， ALOX5， MARCH8， WASHC2C， TIMM23， NCOA4， MSMB， PPYR1， GPRIN2， SYT15， PTPN20， GDF10， GDF2， RBP3， ANXA8， FRMPD2， MAPK8， ARHGAP22， HYPT9， WDFY4， LRRC18， DRGX， ERCC6， CHAT， SLC18A3， OGDHL， PARG， ASAH2， SGMS1， ASAH2B， A1CF， PRKG1， ALL1， ALPQTL4， IBD15， MAFD8， AD037， ARL8， ARMC4， ARTEMIS， ASAH2C， ASP， ATP5C1， BMP3B， BMP9， BMS1L， BS69， C10orf10， C10orf112， C10orf63， C10orf97， CBS1， CD25， CDNF， CFP1， CHDR， CHIF， CKLIK， CKN2， CMS6， CNA43， COPEB， COT， CRFG， CTRCT30， CTRP13， CUGBP2， CX40.1， DDH1， DDH2， DFNB30， DGS2， DMNT2， DRG11， DUBA7， ELE1， FAM21C， FBH1， FLJ14813， FLJ23414， FNRB， GLC1E， GRIN2， HAKRB， HDR， HPC13， HSP70L1， HTJ1， HTSP， IFCR， IFRX， IPFK2， IPPI2， JCAD， KIAA0934， KIAA1074， KIAA1136， KIAA1290， KIAA1294， KIAA1424， KIAA1607， KIAA1617， KIAA1630， KIAA1844， KIAA1996， KIN17， KNS1， KOX15， KOX19， KOX21， KOX30， LARP5， LINC02657， MEN2A， MFM10， MMR， MOK2， MP1， NF11A， NMA， NOCGUS， NPY4R， NRP， OZEMA2， PAC1， PACA， PAHX， PAPD1， PAR3， PI5P4KA， PRKG1B， PRKM8， PRP18， PTPLA， RABGDIB， RED2， RENT2， RHDA1， RIAM， RNTRE， RP66， SCP， SDF1， SIAT8F， SKT， SMS1， SPF45， SPS1， TAFII140， TCF8， TEM7R， TPT， TSH1， VACHT， WARBM3， YME1L， YSA1H， ZIP12， ZNF11B， AAKAD， ASAH2L， ATP5CL1， BCD1， BRAM1， BTCD， CARP， CCAFCA， CCNX， CDR， CILD23， CMS21， CMYP11， COFS1， COQ1， DD1， DD2， DD3， DEPP， DESSH， DIET1， EST， ETR3， FBX18， FIP2， GWL， HDRS， HHT5， HSCR1， IGS1， IL2R， IMD80， IRXL1， JNK1， K100， KIAA0187， KIAA0217， KOX2， NIL2A， PAGEN2， PAMP， PIP5K2A， PP1， PREP， PRKGR1B， PTC3， RARP1， RDLKD， SCIDA， SPAX4， TAKR， THC2， TMEM23， UKHC， VEGF165R， VPEF， YOBELN， ZNF37， AAT8， ACC， ATP5C， BRUNOL3， C10orf9， CMT2Q， COQ10D2， CSB， FIG， HAKRA， HAKRC， HAKRD， HYPL， IDDM10， KIAA0592， MOB， MRD30， OPA11， PPCD3， SAPK1， SCAR30， TPL2， ZF9， ARMD5， DD4， DEE97， FECD6， IMD41， SRXY8， ALS12， UVSS1， POF11 |

Table S8 Common targets of drugs and diseases

| **Pre-screening target** | **After PPI screening** |
| --- | --- |
| NOS2， AR， PTGS2， ESR2， DPP4， NCOA2， F2， KDR， PTGS1， PIK3CG， AKR1B1， JUN， ESR1， PGR， OPRM1， CHRNA7， BCL2， BAX， CASP9， CASP8， CASP3， TGFB1， PON1， MAP2， CDKN1A， EIF6， TNF， TP53， PCNA， MYC， IL1B， CCNB1， PPARG， NCOA1， MMP9， EGF， MMP1， CYP1A1， ACTA2， SLC2A4， RXRA， NOS3 | MMP9， TGFB1， CASP8， EGF， IL1B， PTGS2， CASP3， PPARG， TNF， JUN， AR， TP53， MYC， NOS3， RXRA， NCOA2， NCOA1， ESR |

Table S9 KEGG pathway and screening corresponding to 42 common targets (*p* value<0.05 and delete cancer-related pathways)

| GO | Description | LogP | Enrichment | Z-score | #GeneInHitList | GeneID | Hits | Log(q-value) |
| --- | --- | --- | --- | --- | --- | --- | --- | --- |
| hsa04657 | IL-17 signaling pathway | -11 | 49 | 20 | 42 | 836\|841\|3553\|3725\|4312\|4318\|5743\|7124 | CASP3\|CASP8\|IL1B\|JUN\|MMP1\|MMP9\|PTGS2\|TNF | -8.8 |
| hsa01522 | Endocrine resistance | -11 | 48 | 19 | 42 | 581\|596\|1026\|2099\|2100\|3725\|4318\|7157 | BAX\|BCL2\|CDKN1A\|ESR1\|ESR2\|JUN\|MMP9\|TP53 | -8.7 |
| hsa04933 | AGE-RAGE signaling pathway in diabetic complications | -11 | 46 | 19 | 42 | 581\|596\|836\|3553\|3725\|4846\|7040\|7124 | BAX\|BCL2\|CASP3\|IL1B\|JUN\|NOS3\|TGFB1\|TNF | -8.6 |
| hsa04115 | p53 signaling pathway | -11 | 58 | 20 | 42 | 581\|836\|841\|842\|891\|1026\|7157 | BAX\|CASP3\|CASP8\|CASP9\|CCNB1\|CDKN1A\|TP53 | -8 |
| hsa01524 | Platinum drug resistance | -10 | 55 | 19 | 42 | 581\|596\|836\|841\|842\|1026\|7157 | BAX\|BCL2\|CASP3\|CASP8\|CASP9\|CDKN1A\|TP53 | -7.9 |
| hsa04210 | Apoptosis | -10 | 33 | 16 | 42 | 581\|596\|836\|841\|842\|3725\|7124\|7157 | BAX\|BCL2\|CASP3\|CASP8\|CASP9\|JUN\|TNF\|TP53 | -7.6 |
| hsa04151 | PI3K-Akt signaling pathway | -9.6 | 17 | 12 | 42 | 596\|842\|1026\|1950\|3791\|4609\|4846\|5294\|6256\|7157 | BCL2\|CASP9\|CDKN1A\|EGF\|KDR\|MYC\|NOS3\|PIK3CG\|RXRA\|TP53 | -7.2 |
| hsa04668 | TNF signaling pathway | -9.2 | 37 | 16 | 42 | 836\|841\|3553\|3725\|4318\|5743\|7124 | CASP3\|CASP8\|IL1B\|JUN\|MMP9\|PTGS2\|TNF | -6.8 |
| hsa04919 | Thyroid hormone signaling pathway | -8.9 | 35 | 15 | 42 | 842\|2099\|4609\|6256\|7157\|8648\|10499 | CASP9\|ESR1\|MYC\|RXRA\|TP53\|NCOA1\|NCOA2 | -6.6 |
| hsa04215 | Apoptosis - multiple species | -8.5 | 87 | 21 | 42 | 581\|596\|836\|841\|842 | BAX\|BCL2\|CASP3\|CASP8\|CASP9 | -6.3 |
| hsa04010 | MAPK signaling pathway | -7.9 | 18 | 11 | 42 | 836\|1950\|3553\|3725\|4609\|7040\|7124\|7157 | CASP3\|EGF\|IL1B\|JUN\|MYC\|TGFB1\|TNF\|TP53 | -5.8 |
| hsa04915 | Estrogen signaling pathway | -7.7 | 35 | 14 | 42 | 2099\|2100\|3725\|4318\|4846\|4988 | ESR1\|ESR2\|JUN\|MMP9\|NOS3\|OPRM1 | -5.7 |
| hsa04110 | Cell cycle | -7.1 | 28 | 13 | 42 | 891\|1026\|4609\|5111\|7040\|7157 | CCNB1\|CDKN1A\|MYC\|PCNA\|TGFB1\|TP53 | -5.2 |
| hsa04066 | HIF-1 signaling pathway | -6.1 | 28 | 12 | 42 | 596\|1026\|1950\|4843\|4846 | BCL2\|CDKN1A\|EGF\|NOS2\|NOS3 | -4.3 |
| hsa04380 | Osteoclast differentiation | -5.5 | 22 | 10 | 42 | 3553\|3725\|5468\|7040\|7124 | IL1B\|JUN\|PPARG\|TGFB1\|TNF | -3.8 |
| hsa04068 | FoxO signaling pathway | -5.5 | 22 | 10 | 42 | 891\|1026\|1950\|6517\|7040 | CCNB1\|CDKN1A\|EGF\|SLC2A4\|TGFB1 | -3.8 |
| hsa04370 | VEGF signaling pathway | -5.5 | 39 | 12 | 42 | 842\|3791\|4846\|5743 | CASP9\|KDR\|NOS3\|PTGS2 | -3.8 |
| hsa04921 | Oxytocin signaling pathway | -5.2 | 19 | 9.2 | 42 | 1026\|3725\|4846\|5294\|5743 | CDKN1A\|JUN\|NOS3\|PIK3CG\|PTGS2 | -3.6 |
| hsa01521 | EGFR tyrosine kinase inhibitor resistance | -5 | 29 | 10 | 42 | 581\|596\|1950\|3791 | BAX\|BCL2\|EGF\|KDR | -3.4 |
| hsa04621 | NOD-like receptor signaling pathway | -5 | 17 | 8.7 | 42 | 596\|841\|3553\|3725\|7124 | BCL2\|CASP8\|IL1B\|JUN\|TNF | -3.4 |
| hsa04012 | ErbB signaling pathway | -4.8 | 27 | 10 | 42 | 1026\|1950\|3725\|4609 | CDKN1A\|EGF\|JUN\|MYC | -3.2 |
| hsa04064 | NF-kappa B signaling pathway | -4.6 | 24 | 9.5 | 42 | 596\|3553\|5743\|7124 | BCL2\|IL1B\|PTGS2\|TNF | -3.1 |
| hsa04620 | Toll-like receptor signaling pathway | -4.5 | 22 | 9 | 42 | 841\|3553\|3725\|7124 | CASP8\|IL1B\|JUN\|TNF | -3 |
| hsa04659 | Th17 cell differentiation | -4.4 | 21 | 8.9 | 42 | 3553\|3725\|6256\|7040 | IL1B\|JUN\|RXRA\|TGFB1 | -2.9 |
| hsa04722 | Neurotrophin signaling pathway | -4.3 | 19 | 8.4 | 42 | 581\|596\|3725\|7157 | BAX\|BCL2\|JUN\|TP53 | -2.8 |
| hsa04371 | Apelin signaling pathway | -4 | 17 | 7.7 | 42 | 59\|4843\|4846\|5294 | ACTA2\|NOS2\|NOS3\|PIK3CG | -2.6 |
| hsa04060 | Cytokine-cytokine receptor interaction | -4 | 11 | 6.7 | 42 | 1950\|3553\|3791\|7040\|7124 | EGF\|IL1B\|KDR\|TGFB1\|TNF | -2.6 |
| hsa04920 | Adipocytokine signaling pathway | -3.6 | 25 | 8.3 | 42 | 6256\|6517\|7124 | RXRA\|SLC2A4\|TNF | -2.2 |
| hsa03320 | PPAR signaling pathway | -3.6 | 24 | 8.1 | 42 | 4312\|5468\|6256 | MMP1\|PPARG\|RXRA | -2.2 |
| hsa04510 | Focal adhesion | -3.4 | 12 | 6.2 | 42 | 596\|1950\|3725\|3791 | BCL2\|EGF\|JUN\|KDR | -2.1 |
| hsa04350 | TGF-beta signaling pathway | -3.4 | 21 | 7.5 | 42 | 4609\|7040\|7124 | MYC\|TGFB1\|TNF | -2 |
| hsa04211 | Longevity regulating pathway | -3.3 | 19 | 7.3 | 42 | 581\|5468\|7157 | BAX\|PPARG\|TP53 | -2 |
| hsa04931 | Insulin resistance | -3.1 | 16 | 6.5 | 42 | 4846\|6517\|7124 | NOS3\|SLC2A4\|TNF | -1.7 |
| hsa04725 | Cholinergic synapse | -3 | 15 | 6.4 | 42 | 596\|1139\|5294 | BCL2\|CHRNA7\|PIK3CG | -1.7 |
| hsa04726 | Serotonergic synapse | -3 | 15 | 6.3 | 42 | 836\|5742\|5743 | CASP3\|PTGS1\|PTGS2 | -1.7 |
| hsa04114 | Oocyte meiosis | -2.9 | 14 | 6 | 42 | 367\|891\|5241 | AR\|CCNB1\|PGR | -1.6 |
| hsa04310 | Wnt signaling pathway | -2.7 | 12 | 5.5 | 42 | 3725\|4609\|7157 | JUN\|MYC\|TP53 | -1.4 |
| hsa04630 | Jak-STAT signaling pathway | -2.6 | 11 | 5.3 | 42 | 596\|1026\|4609 | BCL2\|CDKN1A\|MYC | -1.3 |
| hsa04020 | Calcium signaling pathway | -2.4 | 9.5 | 4.8 | 42 | 1139\|4843\|4846 | CHRNA7\|NOS2\|NOS3 | -1.2 |

Table S10:The pathways and function modules of RP treating EMs (Screen the pathways related to these five modules)

| **Pathway** | **Function module** | **Gene** |
| --- | --- | --- |
| IL-17 signaling pathway | Inflammation reaction | CASP3\|CASP8\|IL1B\|JUN\|MMP1\|MMP9\|PTGS2\|TNF |
| Endocrine resistance | Hormone regulation,Proliferation and apoptosis | BAX\|BCL2\|CDKN1A\|ESR1\|ESR2\|JUN\|MMP9\|TP53 |
| AGE-RAGE signaling pathway | Inflammation reaction,Proliferation and apoptosis,Angiogenesis | BAX\|BCL2\|CASP3\|IL1B\|JUN\|NOS3\|TGFB1\|TNF |
| p53 signaling pathway | Proliferation and apoptosis | BAX\|CASP3\|CASP8\|CASP9\|CCNB1\|CDKN1A\|TP53 |
| Platinum drug resistance | Proliferation and apoptosis | BAX\|BCL2\|CASP3\|CASP8\|CASP9\|CDKN1A\|TP53 |
| Apoptosis pathway | Proliferation and apoptosis | BAX\|BCL2\|CASP3\|CASP8\|CASP9\|JUN\|TNF\|TP53 |
| PI3K-Akt signaling pathway | Inflammation reaction,Cell adhesion,Proliferation and apoptosis,Angiogenesis | BCL2\|CASP9\|CDKN1A\|EGF\|KDR\|MYC\|NOS3\|PIK3CG\|RXRA\|TP53 |
| TNF signaling pathway | Inflammation reaction,Cell adhesion | CASP3\|CASP8\|IL1B\|JUN\|MMP9\|PTGS2\|TNF |
| Thyroid hormone signaling pathway | Inflammation reaction,Hormone regulation,Proliferation and apoptosis,Angiogenesis | CASP9\|ESR1\|MYC\|RXRA\|TP53\|NCOA1\|NCOA2 |
| Apoptosis - multiple species | Proliferation and apoptosis | BAX\|BCL2\|CASP3\|CASP8\|CASP9 |
| MAPK signaling pathway | Inflammation reaction,Proliferation and apoptosis | CASP3\|EGF\|IL1B\|JUN\|MYC\|TGFB1\|TNF\|TP53 |
| Estrogen signaling pathway | Hormone regulation | ESR1\|ESR2\|JUN\|MMP9\|NOS3\|OPRM1 |
| Cell cycle | Proliferation and apoptosis | CCNB1\|CDKN1A\|MYC\|PCNA\|TGFB1\|TP53 |
| HIF-1 signaling pathway | Inflammation reaction,Proliferation and apoptosis,Angiogenesis | BCL2\|CDKN1A\|EGF\|NOS2\|NOS3 |
| FoxO signaling pathway | Proliferation and apoptosis | CCNB1\|CDKN1A\|EGF\|SLC2A4\|TGFB1 |
| VEGF signaling pathway | Cell adhesion,Proliferation and apoptosis | CASP9\|KDR\|NOS3\|PTGS2 |
| Oxytocin signaling pathway | Hormone regulation,Proliferation and apoptosis | CDKN1A\|JUN\|NOS3\|PIK3CG\|PTGS2 |
| EGFR tyrosine kinase inhibitor resistance | Proliferation and apoptosis,Angiogenesis | BAX\|BCL2\|EGF\|KDR |
| NOD-like receptor signaling pathway | Inflammation reaction | BCL2\|CASP8\|IL1B\|JUN\|TNF |
| ErbB signaling pathway | Cell adhesion,Proliferation and apoptosis,Angiogenesis | CDKN1A\|EGF\|JUN\|MYC |
| NF-kappa B signaling pathway | Inflammation reaction,Cell adhesion | BCL2\|IL1B\|PTGS2\|TNF |
| Toll-like receptor signaling pathway | Inflammation reaction | CASP8\|IL1B\|JUN\|TNF |
| Th17 cell differentiation | Inflammation reaction | IL1B\|JUN\|RXRA\|TGFB1 |
| Apelin signaling pathway | Proliferation and apoptosis,Angiogenesis | ACTA2\|NOS2\|NOS3\|PIK3CG |
| Cytokine-cytokine receptor interaction | Inflammation reaction,Proliferation and apoptosis,Angiogenesis | EGF\|IL1B\|KDR\|TGFB1\|TNF |
| PPAR signaling pathway | Proliferation and apoptosis | MMP1\|PPARG\|RXRA |
| Focal adhesion | Cell adhesion,Proliferation and apoptosis | BCL2\|EGF\|JUN\|KDR |
| TGF-beta signaling pathway | Proliferation and apoptosis | MYC\|TGFB1\|TNF |
| Insulin resistance | Inflammation reaction | NOS3\|SLC2A4\|TNF |
| Oocyte meiosis | Proliferation and apoptosis | AR\|CCNB1\|PGR |
| Wnt signaling pathway | Cell adhesion,Proliferation and apoptosis | JUN\|MYC\|TP53 |
| Jak-STAT signaling pathway | Proliferation and apoptosis | BCL2\|CDKN1A\|MYC |

|  | **Blank** | **Control** | **1mg/ml** | **3mg/ml** | **5mg/ml** | **7mg/ml** | **9mg/ml** |
| --- | --- | --- | --- | --- | --- | --- | --- |
| **24h** | 0.0526 | 0.8188 | 0.7072 | 0.5595 | 0.3599 | 0.3029 | 0.2408 |
|  | 0.0549 | 0.8205 | 0.7518 | 0.5211 | 0.3235 | 0.2824 | 0.2071 |
|  | 0.0522 | 0.7857 | 0.7224 | 0.5751 | 0.3114 | 0.242 | 0.2397 |
| **48h** | 0.0512 | 1.0448 | 0.7745 | 0.5773 | 0.296 | 0.2176 | 0.2221 |
|  | 0.054 | 1.0713 | 0.7678 | 0.5351 | 0.2817 | 0.2357 | 0.2355 |
|  | 0.0521 | 1.1059 | 0.7385 | 0.535 | 0.2683 | 0.2115 | 0.2646 |

Table S11 MTT result (OD value)

Table S12 Flow cytometry data results

|  | **Early apoptosis** | **Late apoptosis** | **Apoptosis rate** |
| --- | --- | --- | --- |
| **Control group-1** | 2.78% | 1.80% | 4.58% |
| **Control group-2** | 2.88% | 1.67% | 4.55% |
| **Control group-3** | 3.65% | 1.72% | 5.37% |
| **therapy group （3mg/mL）-1** | 25.58% | 14.73% | 40.31% |
| **therapy group （3mg/mL）-2** | 24.76% | 15.45% | 40.21% |
| **therapy group （3mg/mL）-3** | 23.84% | 14.74% | 38.58% |

Table S13:WB result data

|  | Control group-1 | Control group-2 | Control group-3 | therapy group(3mg/Ml)-1 | therapy group(3mg/Ml)-2 | therapy group(3mg/Ml)-3 |
| --- | --- | --- | --- | --- | --- | --- |
| P53 | 114565257 | 129240525 | 107566988 | 189435882 | 193943731 | 203555729 |
| Bax | 51201252 | 71839357 | 65169188 | 122225503 | 132822950 | 130629157 |
| caspase3(32KD) | 124885794 | 114576586 | 117482742 | 118219924 | 113652097 | 113648708 |
| caspase3(17KD) | 40749496 | 35908902 | 48246082 | 86656005 | 90987534 | 93797541 |
| GAPDH | 188893725 | 197645080 | 195381322 | 187971328 | 188888174 | 190386953 |
| P53/GAPDH | 0.607 | 0.654 | 0.551 | 1.008 | 1.027 | 1.069 |
| Bax/GAPDH | 0.271 | 0.363 | 0.334 | 0.650 | 0.703 | 0.686 |
| caspase3(32KD)/GAPDH | 0.661 | 0.580 | 0.601 | 0.629 | 0.602 | 0.597 |
| caspase3(17KD)/GAPDH | 0.216 | 0.182 | 0.247 | 0.461 | 0.482 | 0.493 |


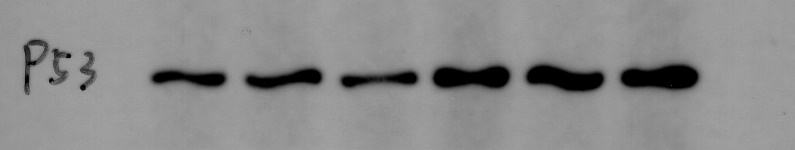


P53(53KDa)


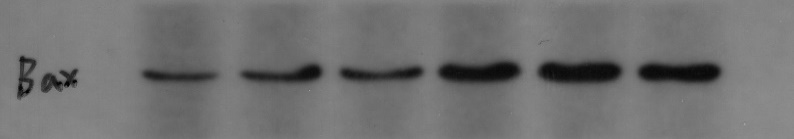


Bax(21KDa)


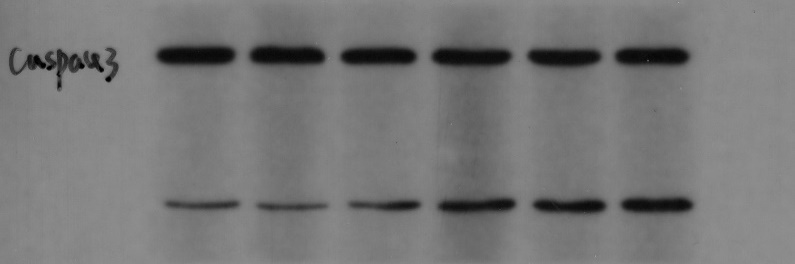


caspase3(32KDa)

caspase3(17KDa)


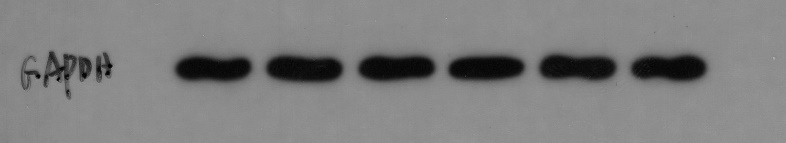


GAPDH(146KDa)

Figure S2 WB strip

Disclaimer: Figure S2 represents a Western Blot (WB) map, and we employed a technique of cutting blots before hybridizing them with antibodies. As illustrated in the figure, the WB assay was performed using three replicate samples for each index. These replicates were obtained from proteins extracted from separate batches of cells. The lacking of visible membrane edges and images of adequate length can be attributed to the simple cropping adjustment applied to the original image, which aims to enhance its aesthetic appeal. It is important to note that the cropping method employed in this image does not undermine the integrity and authenticity of the visual representation.
